# Recent Progress in Atmospheric Chemistry Research in China: Establishing a Theoretical Framework for the “Air Pollution Complex”

**DOI:** 10.1007/s00376-023-2379-0

**Published:** 2023-04-28

**Authors:** Tong Zhu, Mingjin Tang, Meng Gao, Xinhui Bi, Junji Cao, Huizheng Che, Jianmin Chen, Aijun Ding, Pingqing Fu, Jian Gao, Yang Gao, Maofa Ge, Xinlei Ge, Zhiwei Han, Hong He, Ru-Jin Huang, Xin Huang, Hong Liao, Cheng Liu, Huan Liu, Jianguo Liu, Shaw Chen Liu, Keding Lu, Qingxin Ma, Wei Nie, Min Shao, Yu Song, Yele Sun, Xiao Tang, Tao Wang, Tijian Wang, Weigang Wang, Xuemei Wang, Zifa Wang, Yan Yin, Qiang Zhang, Weijun Zhang, Yanlin Zhang, Yunhong Zhang, Yu Zhao, Mei Zheng, Bin Zhu, Jiang Zhu

**Affiliations:** 1 Peking University, Beijing, 100871 China; 2 Guangzhou Institute of Geochemistry, Chinese Academy of Sciences, Guangzhou, 510640 China; 3 Hong Kong Baptist University, Hong Kong SAR, China; 4 Institute of Atmospheric Physics, Chinese Academy of Sciences, Beijing, 100029 China; 5 Chinese Academy of Meteorological Sciences, Beijing, 100081 China; 6 Fudan University, Shanghai, 200438 China; 7 Nanjing University, Nanjing, 210023 China; 8 Tianjin University, Tianjin, 300072 China; 9 Chinese Research Academy of Environmental Sciences, Beijing, 100012 China; 10 Ocean University of China, Qingdao, 266100 China; 11 Institute of Chemistry, Chinese Academy of Sciences, Beijing, 100190 China; 12 Nanjing University of Information Science and Technology, Nanjing, 210044 China; 13 Research Center for Eco-Environmental Sciences, Chinese Academy of Sciences, Beijing, 100085 China; 14 Institute of Earth Environment, Chinese Academy of Sciences, Xi’an, 710061 China; 15 University of Science and Technology of China, Hefei, 230026 China; 16 Tsinghua University, Beijing, 100084 China; 17 Anhui Institute of Optics and Fine Mechanics, Hefei Institutes of Physical Science, Chinese Academy of Sciences, Hefei, 230031 China; 18 Jinan University, Guangzhou, 510632 China; 19 Hong Kong Polytechnic University, Hong Kong SAR, China; 20 Beijing Institute of Technology, Beijing, 100081 China

**Keywords:** atmospheric chemistry, air pollution complex, theoretical framework, recent progress

## Abstract

Atmospheric chemistry research has been growing rapidly in China in the last 25 years since the concept of the “air pollution complex” was first proposed by Professor Xiaoyan TANG in 1997. For papers published in 2021 on air pollution (only papers included in the Web of Science Core Collection database were considered), more than 24 000 papers were authored or co-authored by scientists working in China. In this paper, we review a limited number of representative and significant studies on atmospheric chemistry in China in the last few years, including studies on (1) sources and emission inventories, (2) atmospheric chemical processes, (3) interactions of air pollution with meteorology, weather and climate, (4) interactions between the biosphere and atmosphere, and (5) data assimilation. The intention was not to provide a complete review of all progress made in the last few years, but rather to serve as a starting point for learning more about atmospheric chemistry research in China. The advances reviewed in this paper have enabled a theoretical framework for the air pollution complex to be established, provided robust scientific support to highly successful air pollution control policies in China, and created great opportunities in education, training, and career development for many graduate students and young scientists. This paper further highlights that developing and low-income countries that are heavily affected by air pollution can benefit from these research advances, whilst at the same time acknowledging that many challenges and opportunities still remain in atmospheric chemistry research in China, to hopefully be addressed over the next few decades.

**Article Highlights:**

- Very recent progress in atmospheric chemistry research in China is reviewed.
- Atmospheric chemistry research in China has grown rapidly in the last two decades.
- The "air pollution complex" has evolved from a concept to a theoretical framework.

## 1. Introduction

In 1997, Professor Xiaoyan TANG first proposed the "air pollution complex" concept (Zhu, 2018), building upon her scientific understanding of, and profound insights into, air pollution in China. She pointed out that, due to rapid economic and social developments, urban air pollution in China can be characterized by a combination of the "London Smoke" type (i.e., mainly caused by coal combustion) and the "Los Angeles" type (i.e., photochemical smog mainly caused by vehicle exhaust emissions). This concept reflects the fact that various environmental pollution problems that developed countries experienced in the last century (and perhaps even over a longer period) took place collectively and intensively in one to two decades in fast-developing regions in China. As a result, an air pollution complex, characterized by the co-existence of coal-smoke and photochemical-smog air pollution, appeared (and is still appearing) in many cities in China as a new type of air pollution, and one outstanding feature is its highly complicated formation mechanisms.

The air pollution complex concept opens a new perspective for air pollution control in China, and has been provoking intensive discussion and debates since it was proposed, thereby stimulating important advances in atmospheric chemistry research. A book chapter by Zhu (2005) summarized in brief the scientific understanding of this concept by 2005: rapid urbanization leads to emissions of large amounts of various pollutants into the air, and a myriad of pollutants with high concentrations co-exist in the atmosphere and interact with each other in a complicated manner. What we observe in air pollution complex includes an increase in the atmospheric oxidation capacity, a decrease in atmospheric visibility, and a spread of air quality deterioration over the regional scale. The underlying mechanisms include intricately intertwined sources and sinks of air pollutants, tightly coupled transformation processes of many pollutants, and synergistic or antagonistic effects among various pollutants with respect to their impacts on human and ecosystem health.

Zhu et al. (2011) emphasized that the central part of the air pollution complex concept is that the coexistence of high concentrations of primary and secondary gaseous and particulate pollutants provides a large amount of reactants for heterogeneous reactions. These reactions change the atmospheric oxidation capacity, as well as chemical compositions and physicochemical and optical properties of aerosol particles, thereby accelerating the formation of the air pollution complex.

In 2015, the National Natural Science Foundation of China funded a joint major research program entitled "Formation Mechanisms, Health Effects and Mitigation Strategies of the Air Pollution Complex in China" (Zhu, 2018). This joint research program has two programs, and the first program, entitled "Fundamental Researches on the Formation and Response Mechanism of the Air Pollution Complex in China" (hereafter referred to simply as "the research program"), is led by Tong ZHU and has a total budget of 240 million RMB (2016–23). This research program underscores that not only understanding formation mechanisms of air pollution complexes is a cutting-edge scientific challenge globally, but also its mitigation and control is one of the key national demands in China. It has two major goals: (1) to elucidate the chemical and physical processes critical to the formation of air pollution complexes, to reveal the formation mechanisms of air pollution complexes, and to construct an air pollution complex theoretical framework; and (2) to develop new theories and methodologies for the surveillance, source appointment and decision-making analysis of air pollution complexes, and to propose innovative ideas for controlling air pollution complexes in China.

Thanks to the support of this research program, an air pollution complex theoretical framework has recently been established, and it is of course subject to future development. The main concept of the air pollution complex is that interactions and feedbacks lead to nonlinear relationships between emissions and the level of air pollution (Fig. 1):

(1) Interactions between gaseous molecules and clusters lead to the formation of new particles, and interactions and reactions of molecules and clusters on particle surfaces lead

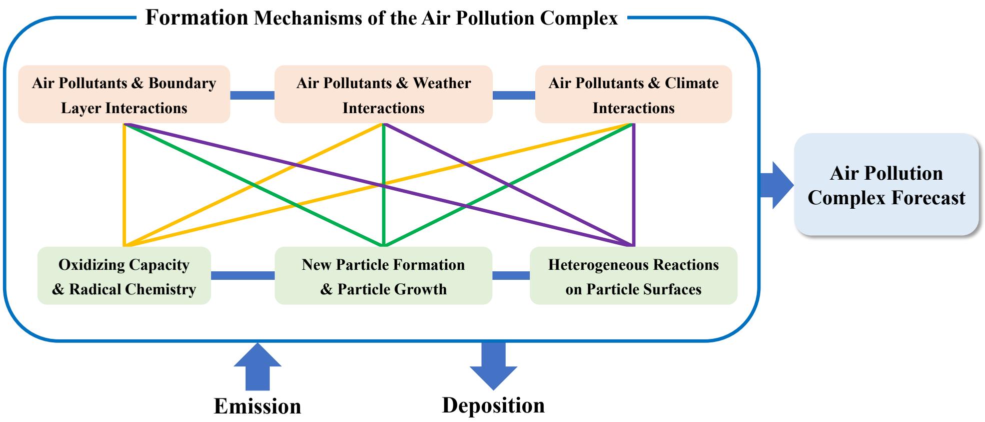

**Fig. 1.** Schematic showing the theoretical framework of the air pollution complex in China.

to the growth of particle sizes. These interaction processes not only result in the formation of secondary aerosols (such as sulfate, nitrate, and secondary organic aerosol), but also contribute to the atmospheric oxidation capacity as radicals and reactive oxygen species are formed via catalytic and photo-enhanced reactions.

(2) Interactions and feedbacks between physical properties and chemical components also play critical roles. Interactions between air pollutants and the planetary boundary layer (PBL) can reduce the boundary layer height, thereby suppressing the dispersion of air pollutants and subsequently enhancing the level of air pollution; and interactions between air pollution and weather as well as climate are larger in terms of spatial and temporal scales, such as the impacts on radiative forcing, cloud formation, and mesoscale and hemispheric circulations (e.g., impacts on monsoons).

The complicated, nonlinear and feedback nature of the air pollution complex can also be described mathematically:

$$\rho(t) = f\{E(t,P), P(t,\rho,C), C(t,E,\rho,P)\},$$

where $\rho(t)$ represents concentrations of air pollutants at the time $t$, $E$ represents the emissions intensity, and $P$ and $C$ are the physical and chemical processes in the atmosphere. $E$, which is a function of anthropogenic activities and natural processes, is also influenced by $P$, such as meteorological factors. There is growing evidence to suggest that concentrations of air pollutants and heterogenous reactions on the surfaces of aerosol particles have important impacts on $P$, such as boundary layer mixing and cloud formation. Finally, it is well established that $C$ is a function of air pollutant concentrations and physical processes in the atmosphere.

Understanding these complicated interactions and feedbacks is essential to better simulate atmospheric physical and chemical processes that lead to the formation of an air pollution complex, to forecast air pollution with much lower uncertainties, and to support air pollution control measures and policies with robust science.

Under the support of this research program and many other funds, atmospheric chemistry research in China has been rapidly growing in various aspects in the last 10–20 years, as illustrated by the following evidence: In the Web of Science Core Collection database, we searched for papers published each year from 2010 to 2021 using the following two sets of keywords (on 31 October 2022): (1) topic: air pollution; address: China; (2) topic: atmospheric chemistry; address: China. As shown in Fig. 2, the number of papers on air pollution and atmospheric chemistry authored or co-authored by scientists working in China has been rapidly and steadily increasing each year in the last decade.

In total, 76 projects have been funded through the aforementioned research program led by Tong ZHU. In order to prepare this review article, the principle investigators of these projects were invited to summarize in brief between one and three important papers (not necessarily their own work) which, in their opinion, represent major progress in atmospheric chemistry research in China in the last two to three years. Their contributions were purely on a voluntary basis, and colleagues who contributed to this review were invited to be co-authors. Clearly, the purpose of this article is not to provide a comprehensive review of all the key progress in atmospheric chemistry research in China in the last few years; rather, it is intended to serve as a starting point for people who want to know more about recent atmospheric chemistry research in China. In this paper, we introduce recent progress in sources and emission inventories (section 2), atmospheric chemical processes (section 3), interactions of air pollution with meteorology, weather and climate (section 4), interactions between the biosphere and atmosphere (section 5), and data assimilation (section 6). In addition, a concise summary and future outlook is provided in section 7.

## 2. Sources and emission inventories

### 2.1. *Emission inventories*

Reliable emission inventories are vital for understanding the sources of air pollution and designing effective measures for air pollution control. As reviewed recently (Li et al., 2017a), major anthropogenic sources in China include

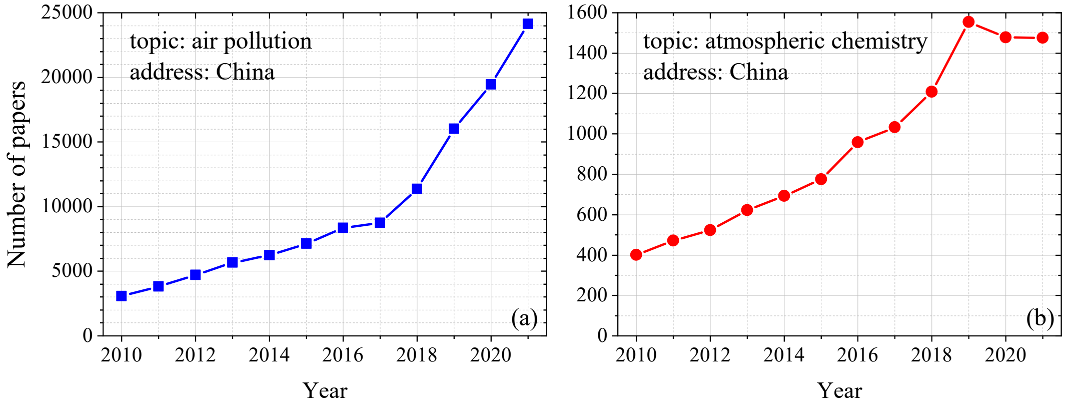

**Fig. 2.** Number of papers published each year from 2010 to 2021 in the Web of Science Core Collection database using two sets of keywords: (a) topic: air pollution; address: China; (b) topic: atmospheric chemistry; address: China.

power plants, industry, residential areas, transportation, solvent use, and agriculture. Here, we review some very recent studies related to emission inventories in China, including transportation emissions (section 2.1.1), volatile organic compounds (VOCs; section 2.1.2), fugitive road dust (section 3.1.3), and methodological advances (section 2.1.4).

### 2.1.1. *Transportation emissions*

Transportation emissions play an important role in the formation of urban air pollution, and ship emissions also contribute to air pollution in coastal cities and marine areas. However, it is highly challenging to estimate emissions from transportation, including ships, due to the complexity and inaccessibility of activity data. With the development of traffic "big data", the technical methods for developing transportation emission inventories have been greatly improved. For truck emissions, Deng et al. (2020) developed a full-sample enumeration approach (TrackATruck), based on 19 billion trajectories, to achieve high-resolution estimation of emission inventories. Their model breaks through the limitations of traditional methods that rely on substitute parameters for spatial and temporal allocation, thus greatly improving the dynamic information density of emission inventories. For ship emissions, Wang et al. (2021e) updated their previous shipping emission inventory model to the second version (SEIM v2.0), which realizes the distinction of emissions from ocean-going, coastal and inland ships, as well as the evaluation of emission reduction benefits of policies.

Maritime transportation accounts for more than 80% of the global trade volume. However, both historical experience and future estimates show that it is rather difficult to achieve significant emission reduction effects by relying on globally collective technological and operational measures. To this end, Wang et al. (2021d) constructed a trade-linked shipping emissions model system (VoySEIM-GTEMS) based on global shipping big data and trade data, and quantitatively decomposed global high-resolution emissions into millions of trade flows, and revealed the heterogeneity of trade emissions efficiency among bilateral trading partners, commodities and shipping routes. An important innovation is that the vessel energy efficiency operational indicator was converted to the trade-emissions efficiency index by value, which enables understanding of the trade-related shipping emissions efficiency at the bilateral level. This model system enables the evaluation of emission reduction efforts from both technical advancement and international trade optimization viewpoints. Thus, it contributes to forming a new shipping emissions reduction framework based on international cooperation, in which contributions from ship owners, operators, and traders can be incorporated and synthetically assessed (Liu et al., 2021a).

### 2.1.2. *VOCs*

Biogenic volatile organic compounds (BVOCs) play vital roles in $O_3$ formation; however, BVOC emissions from urban green spaces have been largely ignored in traditional emission inventories, partly due to the coarse resolution of land cover data. Utilizing land cover data at a spatial resolution of 10 m, Ma et al. (2022) developed a high-resolution (1–27 km) BVOC emissions inventory from urban green spaces (U-BVOC) in China and found that U-BVOC emissions could account for a large fraction (~11%) of the total BVOC emissions in urban cores. It was further found that the addition of U-BVOC emissions could greatly reduce the underestimates of both $O_3$ and its precursors (e.g., isoprene) in Beijing (Ma et al., 2019a; Gao et al., 2022e). This newly developed emissions inventory is publicly available, and continuous assessment is warranted to better understand its impact on air quality in megacities.

Comprehensive gridded emission inventories of semi-volatile/intermediate-volatility organic compounds (S/IVOCs) in China were established based on a parameterization method involving emission factors of primary organic aerosol (POA), emission ratios of S/IVOCs to POA, and domestic activity data (Wu et al., 2019, 2021a). It was found that S/IVOC emissions were mainly distributed in highly industrialized and urbanized regions, with major contributions from industry and residential sectors (Wu et al.,

2021a). The emission inventories of S/IVOCs were further coupled with improved degradation mechanisms and formation schemes of secondary organic aerosol (SOA) in the WRF-Chem model, resulting in a 20% improvement in the resolved fraction of observed SOA (Wu et al., 2019, 2021b). In addition, SOA formation was found to be highly sensitive to several factors, including consideration of SVOCs in the existing POA emissions, configuration of $NO_x$-dependent SOA yields, uncertainties of S/IVOC emissions, and their reaction coefficients with OH radicals.

#### 2.1.3. *Fugitive road dust*

Anthropogenic fugitive, combustion and industrial dust may contribute significantly to global and regional aerosol loadings, but the emission fluxes are not well understood. Chen et al. (2019b) constructed a high-resolution (500 × 500 $m^2$) fugitive road dust $PM_{2.5}$ emissions inventory in Lanzhou (a major city in northwestern China) for the year of 2017. The fugitive road dust $PM_{2.5}$ emission rate, which was estimated to be 1141 ± 71 kg $d^{-1}$, accounted for ~25% of the total $PM_{2.5}$ emission rate in urban Lanzhou (Chen et al., 2019b), and the premature mortality burden due to fugitive road dust $PM_{2.5}$ exposure in Lanzhou was estimated to be 234.5 deaths in 2017. A follow-up study (Xia et al., 2022b) suggested that surface radiative cooling induced by anthropogenic dust, estimated to be up to −15.9 ± 4.0 W $m^{-2}$ regionally, would cause a decrease in boundary layer height and thus deteriorate non-dust air pollution.

#### 2.1.4. *Methodological advances*

Integration of new data from multiple sources has enabled spatial and temporal patterns of emissions to be characterized at finer scales. By using a point source database that includes around 100 000 individual emission facilities, Zheng et al. (2021) developed a new emissions inventory (MEIC-HR) in China with a horizontal resolution of ~1 km. The MEIC-HR dataset significantly improves the spatial representation of the bottom-up emissions inventory in China, with >84% of $SO_2$ emissions and >58% of $NO_x$ emissions presented by point sources. Using MEIC-HR can greatly reduce modeling biases of $PM_{2.5}$ concentrations at 4-km resolution (for example, normalized mean biases were reduced from 27% to 5%).

Zheng et al. (2020a) developed a novel approach to rapidly track dynamic emission changes with sectoral and spatial information by combining bottom-up emission estimates with satellite observations. The bottom-up emissions inventory approach with fast-track statistics was used to provide a preliminary estimate, while a satellite-based inverse modeling approach was developed to infer the 10-day moving average of $NO_x$ emissions, which was then combined with the bottom-up emissions map to constrain dynamic changes of emissions with sectors and grids. The newly developed approach was employed to track dynamic changes of $NO_x$ and $CO_2$ emissions during the COVID-19 lockdown in early 2020 in China (Zheng et al., 2020a), demonstrating the promising prospect of understanding emission dynamics using satellite data.

### 2.2. *Source appointment*

In addition to emission inventories, source appointment based on ambient measurements is a major way to understand sources of air pollution. Zheng et al. (2020b) summarized characteristics of particulate matter pollution in China, and reviewed major methods used to elucidate sources of fine particles. A recent study (Yan et al., 2021) identified combustion to be major sources for brown carbon (BrC) and reactive oxygen species (represented by ·OH radicals) during winter in Beijing, and found that fossil fuel (especially coal) combustion was more important for BrC while biomass burning was more important for reactive oxygen species.

Advanced statistical methods, such as machine learning, have also been employed to understand sources and driving factors of air pollution in China. For example, a machine learning method (the Radom Forest model) was combined with positive matrix factorization to investigate $PM_{2.5}$ pollution in Tianjin between September 2017 and September 2018 (Zhang et al., 2022f), based on online measurements of gaseous and particulate species. It was suggested that source emissions and meteorological conditions contributed 67% and 33% to variations of $PM_{2.5}$ concentrations during the period examined (Zhang et al., 2022f). Furthermore, machine learning has also been used to understand $O_3$ and SOA formation (Wang et al., 2022b) and to elucidate formation pathways of sulfate aerosol (Gao et al., 2022a).

## 3. Atmospheric chemical processes

Our understanding of atmospheric chemical processes relevant to air pollution complexes in China has been substantially improved in the last few years. In this section, we introduce the recent advancements in instrument development (section 3.1), laboratory studies (section 3.2), field observation (section 3.3), and modeling studies (section 3.4).

### 3.1. *Instrument development*

Instrument development plays a critical role in advances in atmospheric chemistry. Atmospheric chemistry research in China has been relying heavily on instruments developed by, and imported from, other countries. Substantial efforts have been devoted to instrument development in China in the past one to two decades, and some recent advances are summarized herein.

#### 3.1.1. *Trace gases*

Amplitude-modulated multimode-diode-laser-based cavity-enhanced absorption spectroscopy, which has the advantages of high light injection efficiency and low cavity-mode noise, was developed to measure $NO_2$ at the wavelength of 406 nm (Zhou et al., 2022), and a detection limit of 8 pptv was achieved with a temporal resolution of 30 s. This technique provides a reliable, simple and self-calibrating method for $NO_2$ measurement, and is especially suitable for long-term operation with minimal maintenance. Furthermore, a custom-built laser-induced fluorescence instrument (AIOFM-LIF) was developed to measure tropospheric OH and $HO_2$ radicals with high sensitivity (Zhang et al., 2022a); it was successfully deployed in several field campaigns, and good agreement with a well-characterized instrument was found (Yang et al., 2021a).

Total OH reactivity, which is equal to the reciprocal of the atmospheric lifetime of OH radicals, is an important parameter to assess atmospheric oxidation capacity. Wei et al. (2020) developed a novel instrument that combined laser-flash photolysis with a mid-infrared Faraday rotation spectrometer for direct measurement of total OH reactivity, and the $1\sigma$ detection precisions at a pressure of 50 mbar were determined to be $4 \times 10^6$ molecule $cm^{-3}$ in 56 s for OH radicals and 0.09 $s^{-1}$ in 112 s for total OH reactivity. Based on magneto-optic effects of paramagnetic species, Faraday rotation spectrometry can effectively reduce spectral interferences caused by diamagnetic precursors, providing a new tool with high precision and high selectivity for OH radical chemistry research. The absorption path length has been further increased by implementing optical-feedback cavity-enhanced absorption spectroscopy technology (Yang et al., 2022b).

### 3.1.2. *Aerosol particles*

Several techniques were recently developed in China to investigate aerosol hygroscopicity, and aerosol hygroscopicity measurement techniques have also been reviewed (Tang et al., 2019a). For example, Zhou et al. (2020) developed a humidified cavity-enhanced albedometer for simultaneous measurement of RH-dependent light extinction, scattering, absorption, and single scattering albedo at 532 nm. It provided a new method for in-situ direct measurement of multiple optical hygroscopic parameters with a single instrument, and aerosol samples could be humidified from 10% to 90% relative humidity (RH) with a cycle time of 15–20 min. Furthermore, Pan et al. (2019) developed an instrument that can measure depolarization ratios of individual particles; it was employed to investigate morphological alteration of aerosol particles caused by heterogeneous chemistry (Pan et al., 2019), and it was found that irregularly shaped particles became more spherical as the mass fraction of water-soluble compositions (such as nitrate) increased up to 8% at high RH.

A series of aerosol liquid water content measurement techniques were developed, including an active RH-controlled dry-ambient aerosol size spectrometer (DAASS), a surface plasmon resonance microscopy (SPRM) imaging system, and a Fourier transform infrared spectroscopy (FTIR) method. The RH-controlled DAASS alternatively measures aerosol size distributions (i.e., 10–500 nm) under dry and ambient conditions, which are then used to quantify the additive volume of aerosol liquid water (Dai et al., 2022). The instrument also employs an active RH controlling scheme and is thus able to minimize the discrepancy between ambient RH and the DAASS conditioning RH (i.e., below 1.5%), extending the application of DAASS to a wide RH range of 10%–90% (Dai et al., 2021b). The SPRM system provides insights into the hygroscopic growth of nano-sized single particles, by allowing imaging of 50-nm polystyrene standard particles with a temporal resolution of 10 ms (Kuai et al., 2020), and thus variations of aerosol water content and refractive index with RH can be derived accordingly. Moreover, hygroscopic growth of nano-sized particles is characterized by reconstructing absorption spectra of aerosol liquid water measured by FTIR, which can also provide information for phase transition dynamics at the molecular level (Wei et al., 2022).

Aerosol acidity impacts numerous physicochemical processes, but the determination of aerosol pH remains a significant challenge due to the non-conservative nature of $H^+$. Cui et al. (2021) developed a direct pH measurement method that uses water as a general probe to detect $H^+$ in individual particles by micro-Raman spectroscopy. The spectra of hydrated ions were decomposed from the solution spectra as standard spectra by multivariate curve resolution analysis, and the concentration profiles of each ion were calculated. This study (Cui et al., 2021) demonstrated that utilizing water, the most common substance, as the spectroscopic probe to measure [$H^+$], has the potential to measure the pH value of atmospheric particles.

Chen et al. (2022c) coupled an aerosol optical tweezer with stimulated Raman spectroscopy to investigate sulfate formation via heterogeneous uptake of $SO_2$ onto aerosol particles. This technique exploits the sensitive size-dependence of stimulated Raman scattering spectra of the droplet to achieve accurate measurement of droplet growth rates, which could be used to derive sulfate formation rates with the knowledge of sulfate hygroscopicity. The detection limit of the droplet radius and sulfate mass can reach 1 nm and $1 \times 10^{-14}$ mol at 60% RH; in addition, this technique also facilitates long experimental times (hours to days) and well-controlled conditions.

Measuring chemical compositions of individual aerosol particles can provide direct evidence for their heterogeneous reactions and mixing states in the atmosphere. Wang et al. (2021b) used micro-Raman spectroscopy to measure chemical compositions of individual particles in aerosol samples collected in Beijing, and $(NH_4)_2SO_4$, $NH_4NO_3$, minerals, carbonaceous materials and $NaNO_3$ were identified according to their characteristic Raman peaks; in addition, they also discussed formation mechanisms of $Ca(NO_3)_2$ and $CaSO_4$ via heterogeneous aging of $CaCO_3$ particles based on their single particle analysis. This technique can directly identify functional groups and molecules in individual aerosol particles under normal ambient conditions (Wang et al., 2021b), rendering it a promising tool for studying coarse particles (>1 μm).

Imidazoles are important photosensitizers in the troposphere, and can impact aerosol optical properties as BrC and have potential risks for human health. In order to measure more imidazoles, Gao et al. (2021b) developed a screening workflow based on data-dependent acquisition auto MS/MS

with a preferred targeted list containing 421 imidazoles, using ultra-performance liquid chromatography quadrupole time-of-flight mass spectrometry (UPLC-QTOF-MS). The method exhibited excellent performance and is able to identify a wide range of imidazoles in ambient aerosol particles with and without using standards.

A versatile aerosol concentration enrichment system (VACES) was developed for hourly measurements of components, ecotoxicity, and optical properties of atmospheric aerosols, significantly increasing detection limits and reducing measurement biases (Shang et al., 2021a), and it has been deployed in field measurements to investigate formation mechanisms of aerosol nitrite (Shang et al., 2021b) and BrC (Kang et al., 2022). For instance, the combination of VACES with ion chromatography and ecotoxicity assays can increase detection limits by one order of magnitude when compared to conventional methods; in addition, integration of VACES with optical instruments can reduce measurement biases by 85% and 47% for light absorption and scattering.

A new dust generation system, which can simulate natural dust emission processes well, was recently developed and verified (Wu et al., 2023). The abundance of water-soluble sulfate in soil-derived dust produced using this system showed good agreement with that found in ambient dust aerosols, supporting the existence of high levels of primary sulfate in dust aerosol emitted from the Taklamakan Desert (Wu et al., 2022). Using the newly developed dust generation system, Wu et al. (2022) further found that aerosol–soil fractionation in the saltation and sandblasting processes could increase water-soluble salts in dust aerosols relative to bulk soil, providing new geochemical constraints for understanding the origins of water-soluble ions in dust aerosol.

## 3.2. *Laboratory studies*

Under the highly complex air pollution conditions in China, strong homogenous nucleation and multiphase processes coexist, coupled with a strong atmospheric oxidation capacity and rapid growth of $PM_{2.5}$. A concept of "haze chemistry" was proposed to reveal the complex air pollution (Chu et al., 2020), which is different from cloud chemistry in London smog and atmospheric photochemistry in Los Angeles photochemical smog. Beyond homogenous processes, gas–liquid–solid multiphase processes are a main issue in haze chemistry.

### 3.2.1. *Inorganic species*

The NO-$NO_2$ cycle determines $O_3$ formation and can enhance heterogeneous and multiphase formation of sulfate, therefore playing a critical role in the tropospheric oxidation capacity. It was found that $SO_2$ can greatly promote heterogeneous transformation of NO into $NO_2$ and HONO on MgO particles under ambient conditions (Liu et al., 2020a). The active sites for adsorption and oxidation of NO were determined to be sulfate, where an intermediate complex, [$SO_4$-NO], was formed during adsorption. The decomposition of [$SO_4$-NO] led to $NO_2$ formation accompanied by the change in sulfate configuration, and the formed $NO_2$ could further react with surface sulfite to generate HONO and sulfate (Ma et al., 2017), thus forming a positive feedback loop (Liu et al., 2020a).

Wang et al. (2021c) carried out environmental chamber experiments under near-ambient conditions and found that the Mn-catalyzed oxidation of $SO_2$ on aerosol surfaces is the dominant pathway for sulfate formation and may explain the missing source of sulfate aerosol. This conclusion was further supported by observation-constrained modeling work (Wang et al., 2022h), which suggested that Mn-catalyzed oxidation on aerosol surfaces could account for >90% of sulfate formation during haze events. For comparison, gas-phase oxidation contributed 3.1% ± 0.5% to sulfate formation due to low OH levels, and $H_2O_2$ oxidation in aerosol water accounted for 4.2% ± 3.6% of sulfate formation because of rapid consumption of $H_2O_2$; contributions of $O_3$ and $NO_2$ oxidation and transition-metal-catalyzed reactions in aerosol water could be negligible owing to low aerosol water contents, low pH, and high ionic strength, while the contribution from in-cloud reactions was negligible due to the barrier caused by stable stratification during winter haze events. We note that several mechanisms have been proposed in the last few years to explain the rapid formation of sulfate aerosol (Cheng et al., 2016; Wang et al., 2016, 2020b; Liu et al., 2020b), and their actual importance is still under debate.

Chen et al. (2021b) investigated the thermodynamics and kinetics of chloride, nitrate and ammonium depletion in atmospheric aerosols. These depletion processes were generalized as a class of unique reactions in which strong acids (HCl and $HNO_3$) and bases ($NH_3$) are irreversibly displaced by weak acids (dicarboxylic acids) and bases (dicarboxylate salts), and these displacement reactions occur exclusively in multiphase aerosols with a large specific surface area that facilitates prompt degassing of volatile species. A subsequent study (Li et al., 2022b) utilized surface-enhanced Raman spectroscopy to measure pH evolution in aerosol microdroplets undergoing ammonium depletion and observed exponential decay of aerosol pH, indicating that a self-limiting feedback mechanism may govern the depletion process.

Tang et al. (2019b) investigated hygroscopic properties of mineral dust samples collected from different regions in northern China using a vapor sorption analyzer (Gu et al., 2017), and found that some mineral dust samples unexpectedly exhibited very high hygroscopicity, due to the presence of soluble ions (such as $Cl^-$, $SO_4^2$ and $Na^+$). A follow-up study (Wang et al., 2022d) found that heterogeneous reactions of $N_2O_5$ with mineral dust could form substantial amounts of $ClNO_2$, and suggested that $N_2O_5$ uptake onto saline mineral dust may be a previously unrecognized but potentially important source of tropospheric $ClNO_2$ in northern China.

Deliquescence and efflorescence RH (DRH and ERH) regulate the phase state and liquid water contents of aerosol particles, and are thus of great interest in atmospheric science and many other fields. Peng et al. (2022a) developed for the first time a comprehensive database that included DRH and ERH values of 110 compounds reported by previous work, provided their preferred DRH and ERH values at 298 K, and identified current knowledge gaps in the deliquescence and efflorescence of atmospheric particles.

### 3.2.2. *Soot particles*

Heterogeneous processes can also lead to the formation of atmospheric radicals, contrary to the conventional view that aerosol particles are always a sink of atmospheric radicals and thereby reduce the atmospheric oxidation capacity. For example, a recent laboratory study (He et al., 2022a) found that the heterogeneous reaction of water and $O_2$ on carbonaceous surfaces could produce gas-phase OH radicals under irradiation, and thus revealed a new formation mechanism of gas-phase OH radicals.

Zhang et al. (2022c) found that diesel soot particles could promote heterogeneous conversion of $SO_2$ to $H_2SO_4$ upon irradiation, and further proved that OH radical is the major reactive oxygen species promoting $SO_2$ oxidation. The photo-electrons from organic carbon react with $O_2$ to produce superoxide radicals ($\cdot O_2^-$), which then combine with $H^+$ ions to form $HO_2$ and $H_2O_2$, and the photolysis of $H_2O_2$ produces OH radical (Zhang et al., 2022c). This work provides new insights into $H_2SO_4$ formation in the atmosphere and also has important implications for assessing health and climate effects of soot particles.

Zhu et al. (2020a) investigated changes in the physicochemical properties and oxidative potential of soot under visible-light irradiation in an environmental chamber with or without $O_3$. Visible light markedly promoted soot oxidation, leading to consumption of polycyclic aromatic hydrocarbons (PAHs), formation of oxygen-containing functional groups, and enhancement of oxidative potential. This study (Zhu et al., 2020a) also suggested that solar irradiation could trigger self-oxidation processes in soot, thereby affecting its atmospheric and health effects. Zhu et al. (2021b) further explored the microstructure, composition and photo-reactivity of soot, and found that the core component of soot to be more analogous to reduced graphene oxide rather than graphene. The generation of reactive oxygen species via electron transfer under visible light indicates that the reduced graphene oxide-like soot core can serve as a potential carbo-photocatalyst (Zhu et al., 2021b).

### 3.2.3. *Organic aerosols*

Using an indoor smog chamber, Yang et al. (2021b) examined the effects of $SO_2$ and $NH_3$ on photo-oxidation of 1,2,4-trimethylbenzene. In the presence of $SO_2$, SOA yields were considerably enhanced due to acid-catalyzed heterogeneous reactions, and a large number of organosulfates were also identified; a similar positive correlation between $NH_3$ levels and SOA formation was also observed. A follow-up study (Yang et al., 2023) further examined the effects of $SO_2$ on ozonolysis of cyclooctene, and suggested that the organosulfates produced through reactions of stabilized Criegee intermediates with $SO_2$ contributed remarkably to SOA formation and growth. Furthermore, SOA formation due to atmospheric oxidation of monocyclic aromatic hydrocarbons (Yang et al., 2022d) and anthropogenic effects on biogenic SOA formation (Xu et al., 2021b) have been reviewed recently.

Shen et al. (2022) found that >70% of highly oxygenated organic molecules (HOMs) unexpectedly formed from minor initial H abstraction channel (~10%) instead of the major OH addition channel in the OH oxidation of $\alpha$-pinene under both low NO (30–100 pptv) and high NO (~20 ppbv) conditions, and thus highlighted that minor reaction pathways can contribute significantly to SOA formation and growth. This work further identified the formation and arrangement of alkoxy radicals as a prerequisite for fast autoxidation and thus the formation of HOMs from $\alpha$-pinene OH oxidation (Shen et al., 2022). This new pathway of HOM formation, generally not considered in current chemical mechanisms, may also be important for OH oxidation of other monoterpenes and cyclic alkenes.

Li et al. (2022e) investigated the aqueous oxidation of eugenol, a typical aromatic compound, and found that the SOA yield could be larger than 100%; in addition, they suggested that aqueous products might be more light-absorptive and more toxic than the precursor (Li et al., 2022e), highlighting the impacts of aqueous SOA formation on air quality and climate. Wang et al. (2022j) investigated the aqueous-phase photolysis of methoxyphenols and nitrophenols in the presence of nitrate, and found that the nitration of methoxyphenols led to enhanced light absorption. It was also shown that organic chromophores, such as methoxyphenols, could in turn promote nitrite formation during nitrate photolysis, likely facilitated by solvated electrons (Wang et al., 2021f); the formed nitrite, either in the aqueous phase or partitioning to the gas phase as HONO, may perturb atmospheric chemistry by generating OH radicals upon photolysis.

## 3.3. ***Field observation***

Recent progress in field measurements in China is introduced in this section, including trace gases (section 3.3.1), aerosol particles (section 3.3.2), and applications of isotopic techniques (section 3.3.3) and remote sensing (section 3.3.4).

### 3.3.1. *Trace gases*

OH radicals dominate daytime atmospheric oxidation processes in the troposphere. A number of field campaigns have been performed in various environments during the past several decades to explore atmospheric oxidation capacities and secondary pollution; however, few of these campaigns were conducted in cold seasons as OH was considered to play a minor role in winter. Three winter campaigns were carried out in Beijing to investigate OH radical chemistry, and revealed a strong atmospheric oxidation capacity that was about one to two times higher than that in European and American urban areas (Tan et al., 2018; Lu et al., 2019; Ma et al., 2019b). The dominant primary source of OH radicals was HONO photolysis during wintertime in Beijing, but a large portion of primary sources were still missing. In addition, Wang et al. (2022c) carried out the first field observation of OH radicals in the Yangtze River Delta (YRD) region, and suggested that monoterpenes could significantly aggravate $O_3$ pollution in this region due to co-occurrence of high $NO_x$ and monoterpenes from anthropogenic activities.

HONO is a crucial precursor of OH radicals, but its formation mechanisms are still controversial. Multi-day measurements of HONO and related pollutants were performed at three heights (8, 120 and 240 m) for the first time in Beijing (Zhang et al., 2020c). HONO concentrations were found to be highest at 120 m, followed by those at 8 m and 240 m. Ground and aerosol surfaces played similar roles in $NO_2$ conversion at 8 m height during the whole measurement period, and $NO_2$ conversion on aerosol surfaces was the most important HONO source aloft during haze days. A strong missing HONO source was found in the urban aloft in the daytime, which was related with solar radiation and consumed OH (Zhang et al., 2020c), thereby suggesting a new formation pathway of HONO in the urban atmosphere.

Cl and Br atoms are potent oxidizers and can strongly influence the abundance of climate- and air quality-relevant trace gases. Previous research in polluted regions was mainly focused on $ClNO_2$ (and to a lesser extent, $Cl_2$), and knowledge of other Cl and Br precursors is very limited. Significant amounts of daytime dihalogen gases were recently observed in China—up to 1 ppbv for $Cl_2$ and 10 pptv for $Br_2$ at a polluted coastal site in Hong Kong (Peng et al., 2022c; Xia et al., 2022a), and up to 60 pptv for BrCl at an inland rural site on the North China Plain (NCP; Peng et al., 2021), suggesting the presence of significant daytime sources. Laboratory experiments showed that photolysis of particulate nitrate under acidic conditions (pH < 3.0) could activate chloride and bromide, accounting for a large fraction of the observed daytime $Cl_2$ and $Br_2$ production in Hong Kong (Peng et al., 2022c; Xia et al., 2022a); in northern China, heavy rural household coal burning during winter and nitrate photolysis led to the elevated daytime BrCl (Peng et al., 2021). After photolysis, these dihalogens would produce Cl and Br atoms, thereby impacting VOC oxidation, $O_3$ production and haze formation (Li et al., 2021b; Peng et al., 2021; Xia et al., 2022a). It was further suggested that nitrate photolysis can also be a significant daytime Cl and Br source in other polluted regions.

The ability in characterizing atmospheric non-methane organic compounds has been significantly improved in recent years using multiple online mass spectrometry methods, and the number of organic compounds identified and quantified can reach >1000 (Wu et al., 2020a; Ye et al., 2021a). Concentrations of oxygenated organic compounds were higher than expected in the Pearl River Delta and NCP regions, comprising ~50% or higher of non-methane organic compounds (Wu et al., 2020a; He et al., 2022b). The "missing" OH reactivity in urban regions was significantly reduced after considering these oxygenated species, the photolysis of which could be a large radical source and thus would affect $O_3$ formation (Wang et al., 2022i).

Wang et al. (2020a) revealed a significant contribution of biomass burning to reactive organic gases in eastern China during the harvest season, via field measurements of a near-complete speciation of reactive organic gases. A follow-up study (Gao et al., 2022d) further quantified the contribution of biomass burning to reactive organic gases, and underscored the importance of household biomass burning in addition to open biomass burning.

### 3.3.2. *Aerosol particles*

A 10-year-long (2011–20) measurement campaign of water-soluble inorganic ions in $PM_{2.5}$ was conducted in Beijing. Due to the implementation of strict air pollution control measures, significant decreases in $PM_{2.5}$ were observed in Beijing, with nitrate, sulfate and ammonium decreasing at 5.10%, 8.80% and 7.64% per year (Wang et al., 2022e), and emission reductions of gaseous precursors, especially $SO_2$, made a large contribution to the reduced $PM_{2.5}$ mass concentrations in Beijing. $PM_{2.5}$ mass concentrations have also experienced a substantial decrease (−9.1% per year) in Nanjing since 2013, accompanied by a larger reduction in $SO_2$ (−16.7% per year) (Ding et al., 2019). In contrast, the nitrate fraction was significantly increased in the cold season (Ding et al., 2019), mainly due to the increased oxidization capacity and increased ammonia availability caused by substantial reductions in $SO_2$.

The change in aerosol chemical compositions with rapidly declining emissions was also well demonstrated during the COVID-19 lockdown. Huang et al. (2021) combined in-situ measurements, satellite observations, and numerical simulations to analyze the nonlinear response of aerosol chemical compositions to emission reductions during the lockdown. A sharp drop in transportation emissions led to a substantial decrease in $NO_x$, and the nonlinear response of $O_3$ to $NO_x$ increased the atmospheric oxidation capacity and subsequently accelerated the formation of secondary aerosols (Ren et al., 2021). Under unfavorable meteorological conditions, faster oxidation offset emission reductions and caused severe haze pollution in eastern China. As a result, a synchronous control of VOCs was proposed as an effective way to overcome deteriorating haze pollution due to $NO_x$ reduction.

Chen et al. (2020a) conducted the first vertical observation of precursors (e.g., $N_2O_5$, HONO and $NO_x$) relevant for aerosol nitrate formation in urban Beijing. A conceptual model was meanwhile developed to understand the spatial and temporal distribution of nitrate formation mechanisms by combining vertical observations and measurements at two other ground sites in urban and suburban Beijing. They found that nitrate formation was mainly driven by OH oxidation in the daytime and by $NO_3$ oxidation at night during the wintertime in Beijing (Chen et al., 2020a).

The first comprehensive vertical measurements of fine particle composition were conducted on a 325 m tower in urban Beijing (Lei et al., 2021; Li et al., 2022f), using a $PM_{2.5}$ time-of-flight aerosol chemical speciation monitor, and it was found that there were significantly larger vertical gradients of aerosol species in winter than summer. In particular, vertical ratios of aqueous-phase to photochemical SOA in winter decreased significantly with height, indicating stronger aqueous-phase chemistry at ground level than the city aloft. Lei et al. (2021) observed large increases in the ratios of most aerosol species at 240 m compared to those at ground level in the early morning in winter, and thus highlighted the impacts of the residual layer on air pollution of the second day. Comparatively, Li et al. (2022f) suggested that aerosol liquid water played a more important role in aerosol formation in summer. Furthermore, it was suggested that the higher nitrate concentration in the city aloft than at ground level during daytime was mainly due to the enhanced gas-particle partitioning driven by aerosol water content and acidity.

Aqueous production of secondary aerosols is a vital but less studied contributor to aerosol pollution and haze events. Wang et al. (2020b) proposed a two-step aqueous-phase sulfate formation pathway based on field observations during winter in Beijing: under fog/cloud conditions, $SO_2$ can be rapidly oxidized by $NO_2$ to form sulfate and $HONO/NO_2^-$, and $HONO/NO_2^-$ will further oxidize $SO_2$ to produce $N_2O$ at a pH of 5.5–7 (due to efficient uptake of ammonia). Moreover, aqueous oxidation of fossil-fuel POA was found to be a major source of SOA during winter in Beijing (Wang et al., 2021a), and ring-breaking oxidation of aromatic species in POA was proposed to be the dominant mechanism, leading to the formation of carbonyls and carboxylic acids.

Low-volatility organic vapors are crucial intermediates that connect VOC oxidation to SOA formation; however, measuring these intermediates poses a considerable challenge owing to their complex compositions and very low concentrations. Detailed measurements of these intermediates, named oxygenated organic molecules (OOMs), were conducted using a Chemical Ionization Atmospheric Pressure Interface Time of Flight Mass Spectrometer in eastern China starting in 2018 (Nie et al., 2022). More than 1500 OOMs were identified and assigned to their likely precursors, with anthropogenic aromatic and aliphatic compounds predominating in winter and non-negligible contributions from biogenic monoterpenes and isoprene found in summer (Liu et al., 2021b; Xu et al., 2021c). The condensation of these OOMs contributed significantly, if not dominantly, to SOA formation, and the unexpected increase in OOM concentrations from very clean to highly polluted environments suggests a positive feedback loop between OOM formation and pollution.

Field measurements (Huang et al., 2020a; Yuan et al., 2020) showed that major light-absorbing species, including methoxyphenols, nitrophenols, PAHs, and oxidized PAHs, could in total account for ~10% of light absorption by organic aerosol. Yuan et al. (2020) further used these light-absorbing species, instead of non-light absorbing organic markers, as inputs in positive matrix factorization analysis to identify sources of light-absorbing organic aerosol in Xi'an. Solid fuel combustion was found to be the dominant source for light-absorbing organic aerosol in winter (~80%), while secondary formation became the main source in summer (~60%).

Wang et al. (2019) developed a black-carbon-tracer method coupled with a statistical approach to separate the light absorption of primary and secondary BrC. In the NCP region, primary emissions were found to contribute more to BrC light absorption than secondary processes, and biomass burning and coal combustion contributed to 60% and 35% of primary BrC absorption at 370 nm (Wang et al., 2019). In contrast, when compared to primary BrC, secondary BrC contributed more to aerosol light absorption in the Tibetan Plateau region (Zhu et al., 2021a).

Single particle analysis has been used in China to understand the formation and aging of aerosol particles. Zhang et al. (2019) performed in-situ measurements of the chemical composition of individual particles across the four seasons in Guangzhou, and observed enhanced aqueous formation of oxalate associated with Fe-containing particles. In addition, in-cloud processes have been found to be an important pathway for the formation of light-absorbing organic nitrogen compounds (Zhang et al., 2020b; Lian et al., 2021; Sun et al., 2021b), such as amines and imidazoles. Using size-resolved single-particle chemical compositions and mixing states, a method for evaluating particle aging was developed and used to investigate aerosol particles in Beijing (Chen et al., 2020b, c). It was suggested that regional transport dominated haze formation when $PM_{2.5}$ was < 100 μg $m^{-3}$, while accumulation of local primary particles and secondary formation prevailed when $PM_{2.5}$ exceeded 100 μg $m^{-3}$.

A quadrupole aerosol chemical speciation monitor and single-particle aerosol mass spectrometry were synchronously deployed to investigate the chemical composition and mixing state of aerosols over the East China Sea (Liu et al., 2020c, 2022d; Sun et al., 2021a). Monomethylamine, trimethylamine, and diethylamine were identified as the most abundant amines, accounting for 50%, 16%, and 29% of amine-containing particles, respectively. Elemental carbon-, K-, Mn-, and Fe-rich particles were abundant in monomethylamine-containing particles, and V-rich particles were abundant in trimethylamine-containing particles, indicating that monomethylamine and trimethylamine mainly originated from terrestrial and harbor anthropogenic sources. For comparison, both terrestrial anthropogenic and marine markers were observed in diethylamine-containing particles, suggesting multiple sources for diethylamine.

Marine aerosols are generally divided into sea spray aerosol (produced via wind–sea surface interaction) and secondary marine aerosol (formed via gas-to-particle formation), and the inability to distinguish between these two aerosol types greatly hinders accurate prediction of the marine radiative balance. Xu et al. (2022b) developed a unique approach to identify sub-micron sea-spray aerosol by using size-resolved hygroscopicity measurements, and found that the number concentrations of sub-micron sea-spray aerosol have been significantly underestimated by traditional methods. In addition, combining field measurements with lab experiments, Huang et al. (2022) found that iodine-initiated heterogeneous chemistry can substantially accelerate particle growth, and this new mechanism may explain the fast growth of marine secondary aerosol.

Fe solubility is one of the major factors affecting health and biogeochemical effects of aerosol Fe. Individual particle analysis (Li et al., 2017b; Zhu et al., 2020b) has shown that nanosized iron oxides from anthropogenic sources can be internally mixed with secondary sulfate/nitrate particles in the atmosphere. Liu et al. (2022b) also found that Fe solubility was correlated positively with aerosol acidity and negatively with particle size (0.32–5.6 μm), as fine iron oxide particles had a longer residence time in the troposphere and larger surface for heterogeneous aging, when compared to coarse particles. Furthermore, Zhang et al. (2022b) observed higher Fe solubility in fine particles than coarse particles, and suggested that primary emissions and secondary formation of dissolved Fe played different roles for Fe solubility enhancement in fine and coarse particles. Very recently, Li et al. (2023) found that leaching solutions and contact time used to extract dissolved aerosol trace metals would greatly influence their measured solubilities, and these effects showed large variations for different trace metals. As a result, in order to increase data comparability, it is warranted to standardize aerosol trace metal solubility measurement protocols.

A series of rain and snow samples in China were examined at the molecular level using Fourier transform ion cyclotron resonance mass spectrometry (Chen et al., 2022a). The derivatives of BVOCs were found to be widely distributed along the Yangtze River Basin and contributed to rainwater dissolved organic matter, and variations in their molecular compositions were influenced by combinations of different climatic, geographical and anthropogenic activities (Chen et al., 2022a). Snow samples from four megacities in North China were analyzed to elucidate the potential "precursor–product" pairs of organic nitrogen substances at the molecular level, revealing that more than 50% of the snow CHON molecules may be related to oxidized and hydrolyzed processes of atmospheric organics (Su et al., 2021). Moreover, a new structural classification was provided for atmospheric organosulfur species using the modified oxygen and redefined aromaticity index, and typical sulfonates and anionic surfactants of anthropogenic origins could be easily distinguished using this method (Su et al., 2022a).

#### 3.3.3. *Application of isotopic techniques*

Measurements of isotopic compositions provide a powerful tool and a new perspective to explore sources and chemical processes of aerosol particles, as demonstrated by a number of recent studies in China, a few examples of which are introduced below.

Fan et al. (2020) compared sulfur isotope fractionation produced in atmospheric sulfate formation via different oxidation processes in Beijing, and found that $SO_2$ oxidized by $O_2$ (catalyzed by transition metal ions) and by $NO_2$ dominated aerosol sulfate formation in a wintertime haze episode. Fan et al. (2022) explored vertical distributions of nitrate formation pathways in Beijing by combining oxygen anomalies ($\Delta^{17}O$) and a Bayesian model. It was found that hydrolysis of $NO_2$ to HONO promoted nitrate production at 120 m height (Fan et al., 2022), and low RH at 260 m height inhibited $N_2O_5$ hydrolysis in the residual layer on winter haze days. Zhang et al. (2022e) carried out 3 h-resolution isotope measurements in Nanjing and revealed that heterogeneous hydrolysis of $N_2O_5$ played an important role in nitrate formation on haze days even during the daytime, while OH oxidation dominated nitrate formation in the clean atmosphere.

Compound-specific dual-carbon isotope ($\delta^{13}C$-$\Delta^{14}C$) analysis was developed to investigate sources and atmospheric chemical processes of organic aerosols (Xu et al., 2021a). A recent study (Xu et al., 2022a) employed this technique to track precursors and the formation of aerosol oxalate, an important component of aqueous SOA. It was found that precursors emitted by anthropogenic activities (e. g., fossil fuel combustion) contributed to >50% of aerosol oxalate (Xu et al., 2022a), in contrast to the traditional view that it originated mainly from biogenic precursors.

Non-conventional isotopic techniques have also been employed in atmospheric chemistry research in China. For example, Fe isotopic compositions have been measured for desert and fly ash samples (Li et al., 2022d) as well as airborne magnetic particulate matter collected in Beijing (Zuo et al., 2022).

#### 3.3.4. *Remote sensing*

Satellite remote sensing from American and European space-borne spectrometers, such as the Tropospheric Monitoring Instrument (TROPOMI) and Ozone Monitoring Instrument (OMI), has been widely used to provide long-term and large-scale information on air pollutants. For instance, Chen et al. (2022b) retrieved kilometer-level glyoxal data from TROPOMI, which were used to identify anthropogenic VOC emission sources.

On the other hand, the poor spectral quality of the Environmental Trace Gases Monitoring Instrument (EMI)—the first Chinese satellite-based ultraviolet-visible spectrometer—makes retrieval of air pollutants difficult. In order to obtain reliable results from EMI, the following optimizations of remote sensing algorithms were conducted: (1) on-orbit wavelength calibration was set up to calculate daily instrumental spectral response functions and wavelength shifts to diminish the fitting residuals; (2) an adaptive iterative retrieval algorithm was developed to select the best retrieval setting with the minimum retrieval uncertainty; and (3) simulated irradiance (instead of measured irradiance) was used to eliminate cross-track stripes in the retrieval. Through these optimizations, global distributions of gaseous pollutants, such as $NO_2$, $SO_2$ and HCHO, were successfully retrieved from EMI and used to locate high-emission spots (Zhang et al., 2020a; Xia et al., 2021; Su et al., 2022b). Furthermore, global air quality variations during the COVID-19 pandemic in early 2020 were evaluated from EMI observations, and the abrupt drop in $NO_2$ for many cities when effective measures were implemented to prevent the spread of the pandemic (Liu et al., 2022a) was successfully captured.

Le et al. (2020) analyzed TROPOMI $NO_2$ data and found substantial reductions in $NO_2$ levels over China during the COVID-19 lockdown period (23 January to 13 February); however, severe $PM_{2.5}$ pollution still occurred in northern China. Observational analysis and modeling work were combined to understand the causes of severe $PM_{2.5}$ pollution during that period (Le et al., 2020), suggested to include enhanced heterogeneous chemistry under high RH, stagnant meteorological conditions, and unaffected power plant and petrochemical industry emissions.

Ozonesonde data and TROPOMI $NO_2$ data were combined with WRF-Chem to investigate the effects of typical strong convection on the vertical redistribution of air pollutants in Nanjing (Zhang et al., 2022d). Ozonesonde observations showed higher $O_3$ and water vapor mixing ratios in the upper troposphere after convection, indicating that strong updrafts transported lower-level air masses into the upper troposphere. Ozone production in the upper troposphere was driven by chemistry (5–10 times the dynamic contribution) and reduced (−40%) by lightning $NO_x$ during the whole convection life cycle. In addition, a new high-resolution retrieval algorithm was developed and employed to estimate the lightning $NO_x$ production efficiency, which was determined to be 60 ± 33 mol $NO_x$ per flash (Zhang et al., 2022d).

Current assessments of aerosol radiative effects still contain large uncertainties, partly because aerosols with complex sources have different shapes, chemical compositions and optical properties. Following the deployment of nationwide ground-based observation networks and the development of satellite remote sensing technology, the knowledge base regarding aerosol chemical, optical and radiative properties over regional and global scales has been significantly improved in recent years. For example, using multi-year observations at 50 sites from the China Aerosol Remote Sensing Network, Che et al. (2019a) characterized the aerosol climatologies for representative remote, rural and urban areas in China, and suggested that coarse particles played a dominant role at rural sites near deserts while light-absorbing fine particles were dominant at most urban sites. Decadal-scale trends in total aerosol loading and aerosol optical depth (AOD) were examined for five aerosol components using multi-angle imaging spectroradiometer retrievals (Gui et al., 2022), and small-sized and spherical aerosols (composed of sulfate, organic matter and black carbon) were found to be the dominant aerosol types driving the interannual variability in land AOD during 2003–18. In addition to anthropogenic and natural emissions, the contribution of meteorological factors to interdecadal changes in regional AOD was found to be non-negligible (Che et al., 2019b). Furthermore, a climatology of concentrations of different aerosol compositions was obtained (Li et al., 2022c) using the newly developed Generalized Retrieval of Atmosphere and Surface Properties component approach.

### 3.4. *Modeling studies*

Recently, the Chinese Earth System Science Numerical Simulator Facility (EarthLab) was successfully constructed, and an integrated air quality modeling system (IAQMS-street), which covers global, regional, urban, and street scales based on a two-way coupling technology, was developed as a key component of EarthLab (Chen et al., 2021a; Wang et al., 2022g). Modules of heterogeneous chemistry, size-resolved aerosol microphysical processes, and mixing states were developed for IAQMS-street to better simulate air pollution in China. Careful comparison with observations has revealed that IAQMS-street can reproduce global and regional aerosol mass and number concentrations reasonably well, and in particular the prediction accuracy of heavy pollution episodes has greatly improved. In addition, a high-resolution online global air quality source-receptor model (GNAQPMS-SM) with an uncertainty analysis tool was developed to effectively compute the contributions of various sources to ambient air pollutants (Ye et al., 2021b, 2023).

Model intercomparison using consistent model inputs is an extremely valuable approach to evaluating the performance of numerical models and understanding the magnitude and sources of model uncertainties. In the last two decades, scientists in China have been actively participating in model intercomparison studies (Han et al., 2008; Wang et al., 2008). Very recently, in the Model Inter-Comparison Study for Asia (MICS-Asia) phase III, scientists in China led model intercomparisons for $NO_2$, CO and $NH_3$ (Kong et al., 2020), $O_3$ and relevant species (Li et al., 2019b), aerosol concentrations (Chen et al., 2019a), reactive nitrogen deposition (Ge et al., 2020), and aerosol radiative effects and feedbacks (Gao et al., 2020).

The heterogeneous uptake of $HO_2$ may be a significant sink of $HO_x$, hence impacting the atmospheric oxidation capacity. A multiphase chemical kinetic box model, PKU-MARK, was developed to simulate heterogeneous reactions of $HO_2$ with aerosol particles based on a novel parameterization of $\gamma(HO_2)$ (Song et al., 2020), and it was found to reproduce well the time series of $\gamma(HO_2)$ reported by a summer field campaign at a rural site. A follow-up study (Song et al., 2022a) suggested that, although $HO_2$ uptake may not change the $O_3$ sensitivity regime classification in a single day, it could reduce net $O_3$ production rates by up to 6 ppbv $h^{-1}$ in the morning.

Surface $O_3$ concentrations steadily increased from 2013 to 2019 (Li et al., 2020b), but emission trends of $NO_x$ and VOCs alone cannot explain well the increase in $O_3$. Based on surface observations and model simulations with GEOS-Chem, Li et al. (2019c) suggested that a decrease in $PM_{2.5}$ could increase summertime $O_3$ over the NCP, due to the role of $PM_{2.5}$ as a scavenger of $HO_2$ radicals that would otherwise react with NO to produce $O_3$. Such an $O_3$ "penalty" was further confirmed by observational evidence that $O_3$ production is suppressed under high $PM_{2.5}$ conditions (Li et al., 2021a). As $O_3$ pollution is less sensitive to $NO_x$ emission controls, the need to regulate VOC emissions was underscored (Li et al., 2019c).

Due to the abrupt drop in $NO_x$ emissions during the COVID-19 lockdown, the maximum daily 8-h average $O_3$ concentrations reached 60–70 ppbv in January 2020 when they were expected to be very low. Using GEOS-Chem simulations, Li et al. (2021a) found that fast $O_3$ production was driven by $HO_x$ radicals from the photolysis of formaldehyde, which was generated by VOC oxidation. It was further suggested that high $O_3$ occurrences could increase in terms of frequency and severity during winter and spring without continuous control to reduce VOC emissions.

An observation-based method was developed to investigate the sensitivity of $O_3$ formation to precursors during two persistent elevated $O_3$ episodes in Guangdong (Song et al., 2022b). Average OH concentrations between 0800 and 1300 (UTC+8), derived using this method, fell into a narrow range (2.5–5.5 × $10^6$ mol $cm^{-3}$) with a weak dependence on $NO_x$, and agreed well with those observed at a rural site in the Pearl River Delta. This method was further used to evaluate $O_3$ production efficiencies, $\varepsilon(NO_x)$ or $\varepsilon$(VOC), defined as the number of $O_3$ molecules produced per molecule of $NO_x$ (or VOC) consumed, and the average $\varepsilon(NO_x)$ and $\varepsilon$(VOC) were determined to be 3.0 and 2.1 ppbv/ppbv, respectively (Song et al., 2022b).

Gao et al. (2021a) combined online measurement data with 3D factor analysis to quantify the contributions of different pathways to secondary inorganic aerosol formation, and identified mixed $NH_4NO_3$ and $(NH_4)_2SO_4$ aqueous processes as the most important pathway during heavy haze events. A solute-strength-dependent thermodynamic and kinetic model was developed and employed to investigate sulfate aerosol formation (Gao et al., 2022a), revealing that aqueous oxidation by $H_2O_2$ was the dominant pathway for sulfate formation, and thus suggesting that target oxidant control could be an effective way to mitigate sulfate aerosol.

By integrating a chemical transport model, nationwide measurements, and a sophisticated ammonia emissions model, Liu et al. (2019) found that controlling ammonia emissions would significantly aggravate acid rain pollution, thus offsetting the benefit of reduced fine particle pollution. As a result, region-specific ammonia control strategies could provide a more rational and effective way to achieve co-benefits in protecting human and ecosystem health in China. They further proposed region-specific emission control strategies in the near future (Liu et al., 2019)—for instance, implementing a reduction in $NH_3$ emissions by 20%–30% in areas with scarce acid rain but heavy fine particle pollution (e.g., northern China), while giving higher priorities to $SO_2$ and $NO_x$ emission controls in current acid rain areas like southern China.

Zhu et al. (2022) investigated the spatiotemporal characteristics of multi-pollutant air pollution over China and attributed the decline in multi-pollution to decreases in $PM_{2.5}$–$PM_{10}$ and $PM_{2.5}$–$O_3$ co-pollution days. In the YRD region, co-pollution days with a maximum daily 8-h average $O_3$ > 160 μg $m^{-3}$ and $PM_{2.5}$ > 75 μg $m^{-3}$ generally occurred under conditions of high RH, low wind speed, and high near-surface air temperature (Dai et al., 2021a). Dai et al. (2023) further found a decreased frequency in $PM_{2.5}$–$O_3$ co-pollution days in the Beijing–Tianjin–Hebei (BTH) region during the warm season (April to October) in 2013–20, but increased proportions of $PM_{2.5}$–$O_3$ co-pollution days in $PM_{2.5}$ pollution days, implying a strengthened relationship between $O_3$ and $PM_{2.5}$ under low-$PM_{2.5}$ conditions (Dai et al., 2023). Based on GEOS-Chem simulations, $PM_{2.5}$–$O_3$ co-pollution days in the BTH region were found to be associated with high concentrations of OH and total oxidants, sulfur oxidation ratio and nitrogen oxidation ratio, with the sulfate concentration ranking top among all aerosol species (Dai et al., 2023).

## 4. Interactions of air pollution with meteorology, weather and climate

PBL meteorology plays a vital role in air quality via modulating the diffusion and transformation of pollutants. Both multi-altitude measurements and atmospheric dynamic–chemistry coupled simulations indicate that turbulent motion shapes the vertical stratification of secondary pollution (Huang et al., 2020c). Enhanced nitrate and sulfate production in the upper PBL and residual layer may contribute substantially to near-surface haze pollution through vertical mixing, underscoring the importance of understanding air pollution in China from a vertical perspective. In turn, aerosols might affect PBL evolution by perturbing the radiative energy balance. Under highly polluted conditions, the attenuation and absorption of incident solar radiation by aerosols can heat the atmosphere and cool the surface, thereby strengthening the inversion layer and deteriorating near-surface air pollution (Wang et al., 2018). Such interactions between aerosols and the PBL could amplify regional haze pollution in eastern China (Huang et al., 2020b).

Besides emission and chemical reactions, the distribution and evolution of air pollutants are also modulated by meteorological conditions. In winter, cold fronts occur periodically and transport air pollutants quickly to downstream regions. Combining observations and tracer-tagged simulations, Kang et al. (2021) described 3D structures of $PM_{2.5}$ during a cold front over eastern China. It was suggested that the strong northwesterly transported aerosol particles from the highly polluted NCP to YRD and transported warm and polluted air mass to the free troposphere along the frontal surface over the YRD. In addition, the contributions of sources in the NCP region to $PM_{2.5}$ in the YRD region increased from ~ 15% to 30% during the cold front. Liu et al. (2022c) further found a seesaw pattern of interannual anomalies of $PM_{2.5}$ between the BTH and YRD regions, and suggested that the low (high) $PM_{2.5}$ difference between the BTH and YRD regions was associated with a strong (weak) East Asian winter monsoon.

An online coupled regional climate–chemistry–aerosol model (RIEMS-Chem) was developed and applied with process analysis to investigate aerosol radiative feedback to haze formation and evolution in the BTH region (Li et al., 2020a). It was found that feedback-induced domain-averaged changes in $PM_{2.5}$ concentrations could reach 45.1 μg $m^{-3}$ (39%) during severe haze episodes (Li et al., 2020a). This feedback effect increases aerosol accumulation in the haze growth stage through weakening vertical diffusion, promoting chemical reactions and enhancing horizontal advection of upwind pollutants; plus, it also enhances removal rates in the dissipation stage, but the effect is weak in the persistence stage.

Gao et al. (2022c) characterized common features of the influence of the aerosol direct radiative effect on meteorology based on five severe $PM_{2.5}$ pollution events in winter during 2013–16. It was found that aerosols caused a significant decrease in radiative flux by 52.1–86.7 W $m^{-2}$, a decrease in 2-m temperature by 0.28°C–0.97°C, and a decrease in PBL height by 23.1–58.5 m (Gao et al., 2022c). Aerosol–radiation interactions, including aerosol–photolysis interactions and aerosol–radiation feedback, can also affect near-surface $O_3$ during co-pollution days. Using WRF-Chem simulations, Yang et al. (2022a) investigated four $PM_{2.5}$–$O_3$ co-pollution episodes that occurred in 2014–17 and found that aerosol–photolysis interactions dominated the reduction in daytime near-surface $O_3$ in North China by inhibiting the chemical production of $O_3$.

Using WRF-Chem coupled with an urban canopy scheme, Wang et al. (2022a) found that local circulation regulated the spatial distribution of aerosols and led to diverse impacts of aerosol radiative effects in urban heat islands. It was further found that adopting cool roofs tends to aggravate $PM_{2.5}$ pollution mostly in lightly polluted regions, indicating that green roofs could be better choices given the current severity of air pollution in China (Wang et al., 2020b). These results may offer valuable information on cooperative management of heat islands and air pollution in China.

The anticyclonic anomalies over northeastern Asia associated with stagnant weather conditions, including weak near-surface winds, temperature inversion in the lower troposphere, low PBL height and high RH, are favorable for haze pollution in the NCP region (Li et al., 2019a). Using a weather classification technique, Li et al. (2022a) identified two conducive patterns with the most occurrences of severe $PM_{2.5}$ pollution (daily $PM_{2.5}$ >150 μg $m^{-3}$), and then linked them to various climate factors. It was suggested that the East Atlantic–West Russia teleconnection pattern and the Victoria Mode of sea surface temperature anomalies, which are found to be the top two dominant climate drivers leading to conducive weather patterns in North China, can be used to predict the frequency of severe $PM_{2.5}$ pollution over North China in the winter (Li et al., 2022a).

Global warming is likely to bring more hot days, which may increase the frequency of $O_3$ pollution days in regions with high anthropogenic emissions. Wang et al. (2022f) suggested that more than half of $O_3$ pollution days during 2014–19 in the NCP region occurred under high-temperature extremes when hot and stable atmospheric conditions enhanced $O_3$ chemical production. Using a Random Forest algorithm combined with GEOS-Chem and CMIP6 climate models, Gong et al. (2022) suggested that future risks of $O_3$ exceedance during hot days would significantly decrease in the 2030s under the SSP1-2.6 scenario, but increase until the 2050s under the SSP5-8.5 scenario.

Zhong et al. (2021) suggested that the 0.8% (10 $yr)^{-1}$ decrease in clouds in China from 1957 to 2005 was primarily caused by global warming, and the moisture–convection–latent-heat feedback cycle was the primary driver of the trends in clouds in China as well as globally. The decreasing trend of clouds in China has important implications for the atmospheric oxidation capacity (Zhong et al., 2021), because enhanced solar insolation as a result of less cloud cover will lead to higher concentrations of OH radicals and $O_3$.

Rapid changes in emissions, such as the unexpected emission reductions during COVID-19 and the stringent emission controls in China since 2013, may affect weather and climate. By using Community Earth System Model version 2 (CESM2) simulations, Yang et al. (2022c) revealed that the dramatic reduction in aerosols during the COVID-19 epidemic in eastern China could have caused a positive sea level pressure anomaly over the northwestern Pacific Ocean, thereby strengthening moisture convergence and contributing to the record rainfall in June–July 2020 in eastern China. Gao et al. (2022b) further employed CESM2 to examine the rapid climate responses to emission reductions in aerosol and $O_3$ precursors over China in 2013–17, and suggested that the increase in $O_3$ and decrease in aerosols in the lower troposphere together resulted in an anomalous warming of 0.16°C ± 0.15°C in eastern China.

## 5. Interactions between the biosphere and atmosphere

The biosphere emits a number of trace gases (such as $N_2O$, $NO_x$ and VOCs) into the atmosphere, affecting $O_3$ and secondary aerosol formation and thus further impacting the radiative balance and climate change. On the other hand, some atmospheric pollutants, such as $O_3$, have direct adverse effects on various plants, the interaction of solar radiation with trace gases and aerosol particles can also impact photosynthesis, and dry and wet deposition is an important sources of nutrients and toxic elements for many ecosystems.

Zhao et al. (2022) combined a generalized additive model and an air quality response surface model to analyze source–sink relationships of sulfur and nitrogen oxides using the ratios of deposition to emissions (D/E). Deposition of sulfate and nitrate was found to decline more slowly than the emissions of their precursors ($SO_2$ and $NO_x$), attributed in part to increased precipitation (Zhao et al., 2022). Furthermore, enhanced transport of air pollution has also played an important role in the rising D/E values in four developed regions of China (Zhao et al., 2022), as has changing aerosol chemistry in the case of sulfur compounds.

Xie et al. (2019) developed a regional climate–chemistry –ecology coupled model (RegCM-Chem-YIBs) that includes a regional climate–chemistry model (RegCM-Chem) and a terrestrial vegetation model (YIBs), which is capable of exploring the interactions among $O_3$, $CO_2$ and $PM_{2.5}$ by simulating interactions between the ecosystem and the atmosphere. Meteorological factors and pollutant concentrations from RegCM-Chem are used to drive YIBs every 6 min, and YIBs simulates physiological processes of vegetation and calculates land surface parameters. Using RegCM-Chem–YIBs, Xie et al. (2019) found that tropospheric $O_3$ had a detrimental effect on plant carbon uptake and led to a greater accumulation of $CO_2$ in the atmosphere, and that the terrestrial carbon sink in China was reduced by 112.2 ± 22.5 TgC $yr^{-1}$ due to $O_3$ damage. A follow-up study (Xie et al., 2020) further suggested that atmospheric aerosols contribute to the terrestrial carbon cycle through diffuse radiation fertilization effects and hydrometeorological feedbacks, and that the current aerosol loading can increase the terrestrial carbon sink in China by 60 TgC $yr^{-1}$.

Methane is an important greenhouse gas that contributes significantly to global warming. Atmospheric OH oxidation is a major sink of methane and thus affects its lifetime and abundance. The methane grown rate in 2020, relative to 2019, was attributed to increased natural emissions and an increased atmospheric lifetime (Peng et al., 2022b). The latter was caused by a decrease in the tropospheric OH concentration by ~1.6% when compared to 2019, mainly due to reduced anthropogenic $NO_x$ emissions associated with the spread of COVID-19. This work provides an interesting example demonstrating that changes in emissions from the biosphere and atmospheric chemical processes both play important roles in determining the abundance of atmospheric species.

## 6. Data assimilation

Chemical data assimilation combines observations of atmospheric composition and chemical transport modeling to improve the accuracy of air quality forecasts, to generate chemical reanalysis datasets, and to constrain emission estimates or other uncertain parameters. Kong et al. (2021) released the first high-resolution Chinese air quality reanalysis dataset (CAQRA), which provides surface fields of $PM_{2.5}$, $PM_{10}$, $SO_2$, $NO_2$, CO and $O_3$ in China between 2013 and 2018 with high spatial (15 km) and temporal (1 h) resolutions. This dataset assimilates observations from more than 1000 surface air quality monitoring sites by using an ensemble Kalman filter and the Nested Air Quality Prediction Modeling System (NAQPMS); in addition, several algorithms have been developed to address the challenges in chemical data assimilations, ensuring the high accuracy of CAQRA. This high accuracy and fine resolution of CAQRA facilitates assessment of long-term human and crop exposure to air pollution and other research related to air quality. A modified ensemble Kalman filter, which addresses filter divergence and enables reuse of costly ensemble simulations, was developed by Wu et al. (2020b) for emission inversions in order to make the process with high temporal and spatial resolutions more affordable. Based on this method, CO and $NO_x$ emissions were inversed with a temporal resolution of one week and a spatial resolution of 5 km (Wu et al., 2020b).

Furthermore, under the support of this research program, a data center for China air pollution complexes has recently been developed and will be released for research community access in 2023.

## 7. Summary and outlook

The air pollution complex concept proposed in 1997 by Professor Xiaoyan TANG was based on the observation that coal combustion and vehicle exhaust emissions coexisted in major cities in China. This concept has led to a paradigm shift in our perspective on the formation mechanisms and control policies of air pollution, with emphasis on complexity, interactions, feedbacks, and nonlinearity of chemical and physical processes in the atmosphere. In the last 25 years, the air pollution complex has evolved from a concept to a comprehensive and sophisticated theoretical framework, thanks to active research on air pollution in China supported by the National Natural Science Foundation of China and many other funding agencies.

Due to the length limitation, we were only able to summarize in this paper a limited number of representative and significant studies in atmospheric chemistry in China in the last couple of years. These advances, together with those not covered in this paper, have enriched the theoretical framework of the air pollution complex and provided air pollution control policies in China with robust scientific support. For example, the work that revealed the important contribution of residential emissions to regional air quality (Liu et al., 2016) has eventually led to large-scale control of residential emissions in northern China via the replacement of domestic usage of coal and biofuels with natural gas and electricity. In addition, the finding that controlling ammonium emissions would mitigate aerosol pollution and nitrogen deposition but aggravate acid rain in some regions (Liu et al., 2019) has not only made policymakers realize the complexity of air pollution in China and empathize with region-specific multipollutant control strategies, but also stimulated ammonia control actions enforced by the ministries of Eco-Environment and Agriculture of China.

Joint efforts from all interested parties in China have led to huge success in air pollution control, and $PM_{2.5}$ mass concentrations in many regions in China have been reduced at unprecedented rates in the last several years. In addition, atmospheric research activities in China have also offered great opportunities in education, training, and career development for many graduate students and young scientists, who have been contributing to, and will undoubtedly make even larger contributions to, advancements in atmospheric chemistry. Although mainly based on research activities in China, the theoretical framework of the air pollution complex is also applicable to other countries and regions in the world. A great number of people in many developing and low-income countries are heavily affected by severe air pollution, and knowledge of the air pollution complex concept and lessons learned from air pollution control in China can help these countries improve their air quality and protect human and ecosystem health, similar to what has been happening in China.

Despite remarkable progress, many challenges remain. The National Ambient Air Quality Standards in China were modified in 2012 to set the annual average $PM_{2.5}$ level to be below 35 μg $m^{-3}$, which is a threshold much higher than the value (5 μg $m^{-3}$) recommended by the World Health Organization in 2021 (Xue et al., 2022). Meanwhile, the significant decrease in $PM_{2.5}$ mass concentrations during the last several years has been accompanied by a slow but steady increase in $O_3$ concentrations in many regions, posing a major challenge for air pollution control in China. Therefore, cost-effective co-control of $PM_{2.5}$ and $O_3$ in China requires further understanding in terms of air pollution complex formation mechanisms. Moreover, China has committed to reach carbon neutrality by 2060, and dramatic changes in atmospheric composition will occur accordingly in the next few decades in China during its journey toward this goal, providing not only a natural laboratory for domestic and international scientists to advance our knowledge in atmospheric chemistry, but also a challenge to coordinate carbon emissions reduction and air quality improvement to achieve the most benefits for human health. Further research is required to address the above-mentioned challenges.

## Author contributions

Tong ZHU conceived the idea behind this paper; Tong ZHU, Mingjin TANG and Meng GAO coordinated the preparation of the paper with contributions from all other authors; except Tong ZHU, Mingjin TANG and Meng GAO, all the other authors are listed alphabetically.

***Acknowledgements***. This work was funded by the National Natural Science Foundation of China (Grant No. 91844000). We would like to thank the IGAC (International Global Atmospheric Chemistry) China Working Group and China Young Scientist Working Group for their input.

***Open Access*** This article is licensed under a Creative Commons Attribution 4.0 International License, which permits use, sharing, adaptation, distribution and reproduction in any medium or format, as long as you give appropriate credit to the original author(s) and the source, provide a link to the Creative Commons licence, and indicate if changes were made. The images or other third party material in this article are included in the article's Creative Commons licence, unless indicated otherwise in a credit line to the material. If material is not included in the article's Creative Commons licence and your intended use is not permitted by statutory regulation or exceeds the permitted use, you will need to obtain permission directly from the copyright holder. To view a copy of this licence, visit http://creativecommons.org/licenses/by/4.0/.

## References

[CR1] Che H Z (2019). Large contribution of meteorological factors to inter-decadal changes in regional aerosol optical depth. Atmospheric Chemistry and Physics.

[CR2] Che H Z (2019). Spatial distribution of aerosol microphysical and optical properties and direct radiative effect from the China Aerosol Remote Sensing Network. Atmospheric Chemistry and Physics.

[CR3] Chen L (2019). MICS-Asia III: Multi-model comparison and evaluation of aerosol over East Asia. Atmospheric Chemistry and Physics.

[CR4] Chen S (2022). Source and formation process impact the chemodiversity of rainwater dissolved organic matter along the Yangtze River Basin in summer. Water Research.

[CR5] Chen S Y (2019). Fugitive road dust PM_2.5_ emissions and their potential health impacts. Environ. Sci. Technol..

[CR6] Chen X S (2021). Global–regional nested simulation of particle number concentration by combing microphysical processes with an evolving organic aerosol module. Atmospheric Chemistry and Physics.

[CR7] Chen X R (2020). Field determination of nitrate formation pathway in winter Beijing. Environ. Sci. Technol..

[CR8] Chen Y (2020). Simultaneous measurements of urban and rural particles in Beijing–Part 2: Case studies of haze events and regional transport. Atmospheric Chemistry and Physics.

[CR9] Chen Y (2020). Simultaneous measurements of urban and rural particles in Beijing–Part 1: Chemical composition and mixing state. Atmospheric Chemistry and Physics.

[CR10] Chen Y J (2022). Kilometer-level glyoxal retrieval via satellite for anthropogenic volatile organic compound emission source and secondary organic aerosol formation identification. Remote Sensing of Environment.

[CR11] Chen Z, Liu P, Liu Y, Zhang Y-H (2021). Strong acids or bases displaced by weak acids or bases in aerosols: Reactions driven by the continuous partitioning of volatile products into the gas phase. Accounts of Chemical Research.

[CR12] Chen Z, Liu P, Wang W G, Cao X, Liu Y-X, Zhang Y-H, Ge M F (2022). Rapid sulfate formation via uncatalyzed autoxidation of sulfur dioxide in aerosol microdroplets. Environ. Sci. Technol..

[CR13] Cheng Y F (2016). Reactive nitrogen chemistry in aerosol water as a source of sulfate during haze events in China. Science Advances.

[CR14] Chu B W, Ma Q X, Duan F K, Ma J Z, Jiang J K, He K B, He H (2020). Atmospheric “Haze Chemistry”: Concept and research prospects. Progress in Chemistry.

[CR15] Cui X Y, Tang M J, Wang M J, Zhu T (2021). Water as a probe for pH measurement in individual particles using micro-Raman spectroscopy. Analytica Chimica Acta.

[CR16] Dai H B, Zhu J, Liao H, Li J D, Liang M X, Yang Y, Yue X (2021). Co-occurrence of ozone and PM_2.5_ pollution in the Yangtze River Delta over 2013–2019: Spatiotemporal distribution and meteorological conditions. Atmospheric Research.

[CR17] Dai H B (2023). Composited analyses of the chemical and physical characteristics of co-polluted days by ozone and PM_2.5_ over 2013–2020 in the Beijing–Tianjin–Hebei region. Atmospheric Chemistry and Physics.

[CR18] Dai H S, Gui H Q, Zhang J S, Wei X L, Xie Z B, Bian J J, Chen D-R, Liu J G (2021). An active RH-controlled dry-ambient aerosol size spectrometer (DAASS) for the accurate measurement of ambient aerosol water content. Journal of Aerosol Science.

[CR19] Dai H S (2022). Characteristics of aerosol size distribution and liquid water content under ambient RH conditions in Beijing. Atmos. Environ..

[CR20] Deng F Y, Lv Z F, Qi L J, Wang X T, Shi M S, Liu H (2020). A big data approach to improving the vehicle emission inventory in China. Nature Communications.

[CR21] Ding A J (2019). Significant reduction of PM_2.5_ in eastern China due to regional-scale emission control: Evidence from SORPES in 2011–2018. Atmospheric Chemistry and Physics.

[CR22] Fan M-Y (2020). Roles of sulfur oxidation pathways in the variability in stable sulfur isotopic composition of sulfate aerosols at an urban site in Beijing, China. Environmental Science & Technology Letters.

[CR23] Fan M-Y (2022). Important role of NO_3_ radical to nitrate formation aloft in urban Beijing: Insights from triple oxygen isotopes measured at the tower. Environ. Sci. Technol..

[CR24] Gao J, Shi G L, Zhang Z C, Wei Y T, Tian X, Feng Y C, Russell A G, Nenes A (2022). Targeting atmospheric oxidants can better reduce sulfate aerosol in China: H_2_O_2_ aqueous oxidation pathway dominates sulfate formation in haze. Environ. Sci. Technol..

[CR25] Gao J (2021). Impact of formation pathways on secondary inorganic aerosol during haze pollution in Beijing: Quantitative evidence from high-resolution observation and modeling. Geophys. Res. Lett..

[CR26] Gao J Y (2022). Fast climate responses to emission reductions in aerosol and ozone precursors in China during 2013–2017. Atmospheric Chemistry and Physics.

[CR27] Gao K, Zhang Y D, Liu Y Y, Yang M G, Zhu T (2021). Screening of imidazoles in atmospheric aerosol particles using a hybrid targeted and untargeted method based on ultra-performance liquid chromatography-quadrupole time-of-flight mass spectrometry. Analytica Chimica Acta.

[CR28] Gao M (2020). Air quality and climate change, Topic 3 of the Model Inter-Comparison Study for Asia Phase III (MICS-Asia III)–Part 2: Aerosol radiative effects and aerosol feedbacks. Atmospheric Chemistry and Physics.

[CR29] Gao Y (2022). Impacts of biogenic emissions from urban landscapes on summer ozone and secondary organic aerosol formation in megacities. Science of the Total Environment.

[CR30] Gao Y C, Liao H, Chen H S, Zhu B, Hu J L, Ge X L, Chen L, Li J D (2022). Composite analysis of aerosol direct radiative effects on meteorology during wintertime severe haze events in the North China Plain. J. Geophys. Res.: Atmos.

[CR31] Gao Y Q (2022). Unexpected high contribution of residential biomass burning to non-methane organic gases (NMOGs) in the Yangtze River Delta region of China. J. Geophys. Res.: Atmos.

[CR32] Ge B (2020). Model Inter-Comparison Study for Asia (MICS-Asia) phase III: Multimodel comparison of reactive nitrogen deposition over China. Atmospheric Chemistry and Physics.

[CR33] Gong C, Wang Y, Liao H, Wang P Y, Jin J B, Han Z W (2022). Future co-occurrences of hot days and ozone-polluted days over China under scenarios of shared socioeconomic pathways predicted through a machine-learning approach. Earth’s Future.

[CR34] Gu W J (2017). Investigation of water adsorption and hygroscopicity of atmospherically relevant particles using a commercial vapor sorption analyzer. Atmospheric Measurement Techniques.

[CR35] Gui K (2022). The significant contribution of small-sized and spherical aerosol particles to the decreasing trend in total aerosol optical depth over land from 2003 to 2018. Engineering.

[CR36] Han Z (2008). MICS-Asia II: Model intercomparison and evaluation of ozone and relevant species. Atmos. Environ..

[CR37] He G Z (2022). Generation and release of OH radicals from the reaction of H_2_O with O_2_ over soot. Angewandte Chemie International Edition.

[CR38] He X J (2022). Volatile organic compounds in wintertime North China Plain: Insights from measurements of proton transfer reaction time-of-flight mass spectrometer (PTR-ToF-MS). Journal of Environmental Sciences.

[CR39] Huang R-J (2020). Water-insoluble organics dominate brown carbon in wintertime urban aerosol of China: Chemical characteristics and optical properties. Environ. Sci. Technol..

[CR40] Huang R-J (2022). Heterogeneous iodine-organic chemistry fast-tracks marine new particle formation. Proceedings of the National Academy of Sciences of the United States of America.

[CR41] Huang X, Ding A J, Wang Z L, Ding K, Gao J, Chai F H, Fu C B (2020). Amplified transboundary transport of haze by aerosol–boundary layer interaction in China. Nature Geoscience.

[CR42] Huang X (2020). Chemical boundary layer and its impact on air pollution in northern China. Environmental Science & Technology Letters.

[CR43] Huang X (2021). Enhanced secondary pollution off-set reduction of primary emissions during COVID-19 lockdown in China. National Science Review.

[CR44] Kang H H (2022). Accurate observation of black and brown carbon in atmospheric fine particles via a versatile aerosol concentration enrichment system (VACES). Science of the Total Environment.

[CR45] Kang H Q (2021). Three-dimensional distribution of PM_2.5_ over the Yangtze River Delta as cold fronts moving through. J. Geophys. Res.: Atmos.

[CR46] Kong L (2020). Evaluation and uncertainty investigation of the NO_2_, CO and NH_3_ modeling over China under the framework of MICS-Asia III. Atmospheric Chemistry and Physics.

[CR47] Kong L (2021). A 6-year-long (2013–2018) high-resolution air quality reanalysis dataset in China based on the assimilation of surface observations from CNEMC. Earth System Science Data.

[CR48] Kuai Y (2020). Real-time measurement of the hygroscopic growth dynamics of single aerosol nanoparticles with bloch surface wave microscopy. ACS Nano.

[CR49] Le T H, Wang Y, Liu L, Yang J N, Yung Y L, Li G H, Seinfeld J H (2020). Unexpected air pollution with marked emission reductions during the COVID-19 outbreak in China. Science.

[CR50] Lei L (2021). Vertical distributions of primary and secondary aerosols in urban boundary layer: Insights into sources, chemistry, and interaction with meteorology. Environ. Sci. Technol..

[CR51] Li J (2019). Model evaluation and intercomparison of surface-level ozone and relevant species in East Asia in the context of MICS-Asia Phase III–Part 1: Overview. Atmospheric Chemistry and Physics.

[CR52] Li J D, Liao H, Hu J L, Li N (2019). Severe particulate pollution days in China during 2013–2018 and the associated typical weather patterns in Beijing-Tianjin-Hebei and the Yangtze River Delta regions. Environmental Pollution.

[CR53] Li J D (2022). Winter particulate pollution severity in North China driven by atmospheric teleconnections. Nature Geoscience.

[CR54] Li J W, Han Z W, Wu Y F, Xiong Z, Xia X G, Li J, Liang L, Zhang R J (2020). Aerosol radiative effects and feedbacks on boundary layer meteorology and PM_2.5_ chemical components during winter haze events over the Beijing-Tianjin-Hebei region. Atmospheric Chemistry and Physics.

[CR55] Li K, Jacob D J, Shen L, Lu X, De Smedt I, Liao H (2020). Increases in surface ozone pollution in China from 2013 to 2019: Anthropogenic and meteorological influences. Atmospheric Chemistry and Physics.

[CR56] Li K (2019). A two-pollutant strategy for improving ozone and particulate air quality in China. Nature Geoscience.

[CR57] Li K (2021). Ozone pollution in the North China Plain spreading into the late-winter haze season. Proceedings of the National Academy of Sciences of the United States of America.

[CR58] Li L (2022). Climatology of aerosol component concentrations derived from multi-angular polarimetric POLDER-3 observations using GRASP algorithm. Earth System Science Data.

[CR59] Li L-F, Chen Z, Liu P, Zhang Y-H (2022). Direct measurement of pH evolution in aerosol microdroplets undergoing ammonium depletion: A surface-enhanced raman spectroscopy approach. Environ. Sci. Technol..

[CR60] Li M (2017). Anthropogenic emission inventories in China: A review. National Science Review.

[CR61] Li Q Y (2021). Halogens enhance haze pollution in China. Environ. Sci. Technol..

[CR62] Li R (2022). Mass fractions, solubility, speciation and isotopic compositions of iron in coal and municipal waste fly ash. Science of the Total Environment.

[CR63] Li R (2023). Evaluating the effects of contact time and leaching solution on measured solubilities of aerosol trace metals. Applied Geochemistry.

[CR64] Li W J (2017). Air pollution–aerosol interactions produce more bioavailable iron for ocean ecosystems. Science Advances.

[CR65] Li X D (2022). Optical and chemical properties and oxidative potential of aqueous-phase products from OH and ^3^C*-initiated photooxidation of eugenol. Atmospheric Chemistry and Physics.

[CR66] Li Y (2022). Vertically resolved aerosol chemistry in the low boundary layer of Beijing in summer. Environ. Sci. Technol..

[CR67] Lian X F (2021). Evidence for the formation of imidazole from carbonyls and reduced nitrogen species at the individual particle level in the ambient atmosphere. Environmental Science & Technology Letters.

[CR68] Liu C (2020). Efficient conversion of NO to NO_2_ on SO_2_-aged MgO under atmospheric conditions. Environ. Sci. Technol..

[CR69] Liu C (2022). First Chinese ultraviolet–visible hyperspectral satellite instrument implicating global air quality during the COVID-19 pandemic in early 2020. Light: Science & Applications.

[CR70] Liu H, Kaewunruen S, Ahmad W N K, Shamsuddin A, Ayetor G K, Hansson J, Bräunl T (2021). A net-zero future for freight. One Earth.

[CR71] Liu J (2016). Air pollutant emissions from Chinese households: A major and underappreciated ambient pollution source. Proceedings of the National Academy of Sciences of the United States of America.

[CR72] Liu L (2022). Size-dependent aerosol iron solubility in an urban atmosphere. NPJ Climate and Atmospheric Science.

[CR73] Liu M X (2019). Ammonia emission control in China would mitigate haze pollution and nitrogen deposition, but worsen acid rain. Proceedings of the National Academy of Sciences of the United States of America.

[CR74] Liu T Y, Clegg S L, Abbatt J P D (2020). Fast oxidation of sulfur dioxide by hydrogen peroxide in deliquesced aerosol particles. Proceedings of the National Academy of Sciences of the United States of America.

[CR75] Liu X H, Zhu B, Zhu T, Liao H (2022). The seesaw pattern of PM_2.5_ interannual anomalies between Beijing-Tianjin-Hebei and Yangtze River Delta across eastern China in winter. Geophys. Res. Lett..

[CR76] Liu Y L (2021). Formation of condensable organic vapors from anthropogenic and biogenic volatile organic compounds (VOCs) is strongly perturbed by NO_x_ in eastern China. Atmospheric Chemistry and Physics.

[CR77] Liu Z (2020). Size-resolved mixing states and sources of amine-containing particles in the East China Sea. J. Geophys. Res.: Atmos.

[CR78] Liu Z (2022). Real-time single particle characterization of oxidized organic aerosols in the East China Sea. npj Climate and Atmospheric Science.

[CR79] Lu K D (2019). Fast photochemistry in wintertime haze: Consequences for pollution mitigation strategies. Environ. Sci. Technol..

[CR80] Ma M C (2019). Substantial ozone enhancement over the North China Plain from increased biogenic emissions due to heat waves and land cover in summer 2017. Atmospheric Chemistry and Physics.

[CR81] Ma M C (2022). Development and assessment of a high-resolution biogenic emission inventory from urban green spaces in China. Environ. Sci. Technol..

[CR82] Ma Q X, Wang T, Liu C, He H, Wang Z, Wang W H, Liang Y T (2017). SO_2_ initiates the efficient conversion of NO_2_ to HONO on MgO surface. Environ. Sci. Technol..

[CR83] Ma X F (2019). Winter photochemistry in Beijing: Observation and model simulation of OH and HO_2_ radicals at an urban site. Science of the Total Environment.

[CR84] Nie W (2022). Secondary organic aerosol formed by condensing anthropogenic vapours over China’s megacities. Nature Geoscience.

[CR85] Pan X L (2019). Synergistic effect of water-soluble species and relative humidity on morphological changes in aerosol particles in the Beijing megacity during severe pollution episodes. Atmospheric Chemistry and Physics.

[CR86] Peng C, Chen L X D, Tang M J (2022). A database for deliquescence and efflorescence relative humidities of compounds with atmospheric relevance. Fundamental Research.

[CR87] Peng S S (2022). Wetland emission and atmospheric sink changes explain methane growth in 2020. Nature.

[CR88] Peng X (2021). An unexpected large continental source of reactive bromine and chlorine with significant impact on wintertime air quality. National Science Review.

[CR89] Peng X (2022). Photodissociation of particulate nitrate as a source of daytime tropospheric Cl_2_. Nature Communications.

[CR90] Ren C H (2021). Nonlinear response of nitrate to NO_x_ reduction in China during the COVID-19 pandemic. Atmos. Environ..

[CR91] Shang X N (2021). A semicontinuous study on the ecotoxicity of atmospheric particles using a versatile aerosol concentration enrichment system (VACES): Development and field characterization. Atmospheric Measurement Techniques.

[CR92] Shang X N (2021). PM_1.0_-nitrite heterogeneous formation demonstrated via a modified versatile aerosol concentration enrichment system coupled with ion chromatography. Environ. Sci. Technol..

[CR93] Shen H R, Vereecken L, Kang S, Pullinen I, Fuchs H, Zhao D F, Mentel T F (2022). Unexpected significance of a minor reaction pathway in daytime formation of biogenic highly oxygenated organic compounds. Science Advances.

[CR94] Song H, Lu K D, Dong H B, Tan Z F, Chen S Y, Zeng L M, Zhang Y H (2022). Reduced aerosol uptake of hydroperoxyl radical may increase the sensitivity of ozone production to volatile organic compounds. Environmental Science & Technology Letters.

[CR95] Song H (2020). Influence of aerosol copper on HO_2_ uptake: A novel parameterized equation. Atmospheric Chemistry and Physics.

[CR96] Song K X (2022). Observation-based analysis of ozone production sensitivity for two persistent ozone episodes in Guangdong, China. Atmospheric Chemistry and Physics.

[CR97] Su S H (2021). High molecular diversity of organic nitrogen in urban snow in North China. Environ. Sci. Technol..

[CR98] Su S H (2022). A new structural classification scheme for dissolved organic sulfur in urban snow from North China. Environmental Science & Technology Letters.

[CR99] Su W J (2022). First global observation of tropospheric formaldehyde from Chinese GaoFen-5 satellite: Locating source of volatile organic compounds. Environmental Pollution.

[CR100] Sun J F (2021). Secondary inorganic ions characteristics in PM_2.5_ along offshore and coastal areas of the megacity Shanghai. J. Geophys. Res.: Atmos.

[CR101] Sun W (2021). Measurement report: Molecular characteristics of cloud water in southern China and insights into aqueous-phase processes from Fourier transform ion cyclotron resonance mass spectrometry. Atmospheric Chemistry and Physics.

[CR102] Tan Z F (2018). Wintertime photochemistry in Beijing: Observations of RO_x_ radical concentrations in the North China Plain during the BEST-ONE campaign. Atmospheric Chemistry and Physics.

[CR103] Tang M J (2019). A review of experimental techniques for aerosol hygroscopicity studies. Atmospheric Chemistry and Physics.

[CR104] Tang M J (2019). Hygroscopic properties of saline mineral dust from different regions in China: Geographical variations, compositional dependence, and atmospheric implications. J. Geophys. Res.: Atmos.

[CR105] Wang F, Carmichael G R, Wang J, Chen B, Huang B, Li Y G, Yang Y J, Gao M (2022). Circulation-regulated impacts of aerosol pollution on urban heat island in Beijing. Atmospheric Chemistry and Physics.

[CR106] Wang F (2022). Machine learning and theoretical analysis release the non-linear relationship among ozone, secondary organic aerosol and volatile organic compounds. Journal of Environmental Sciences.

[CR107] Wang G H (2016). Persistent sulfate formation from London Fog to Chinese haze. Proceedings of the National Academy of Sciences of the United States of America.

[CR108] Wang H C (2022). Anthropogenic monoterpenes aggravating ozone pollution. National Science Review.

[CR109] Wang H C (2022). N_2_O_5_ uptake onto saline mineral dust: A potential missing source of tropospheric ClNO_2_ in inland China. Atmospheric Chemistry and Physics.

[CR110] Wang H L (2020). Atmospheric processing of nitrophenols and nitrocresols from biomass burning emissions. J. Geophys. Res.: Atmos.

[CR111] Wang J F (2020). Fast sulfate formation from oxidation of SO_2_ by NO_2_ and HONO observed in Beijing haze. Nature Communications.

[CR112] Wang J F (2021). Aqueous production of secondary organic aerosol from fossil-fuel emissions in winter Beijing haze. Proceedings of the National Academy of Sciences of the United States of America.

[CR113] Wang J Q, Gao J, Che F, Wang Y L, Lin P C, Zhang Y C (2022). Decade-long trends in chemical component properties of PM_2.5_ in Beijing, China (2011–2020). Science of the Total Environment.

[CR114] Wang M J, Zheng N, Zhao D F, Shang J, Zhu T (2021). Using micro-raman spectroscopy to investigate chemical composition, mixing states, and heterogeneous reactions of individual atmospheric particles. Environ. Sci. Technol..

[CR115] Wang P Y (2022). North China Plain as a hot spot of ozone pollution exacerbated by extreme high temperatures. Atmospheric Chemistry and Physics.

[CR116] Wang Q Y (2019). Wintertime optical properties of primary and secondary brown carbon at a regional site in the North China plain. Environ. Sci. Technol..

[CR117] Wang T (2022). An integrated air quality modeling system coupling regional-urban and street models in Beijing. Urban Climate.

[CR118] Wang T T (2022). Sulfate formation apportionment during winter haze events in North China. Environ. Sci. Technol..

[CR119] Wang W G (2021). Sulfate formation is dominated by manganese-catalyzed oxidation of SO_2_ on aerosol surfaces during haze events. Nature Communications.

[CR120] Wang W J (2022). Direct observations indicate photodegradable oxygenated volatile organic compounds (OVOCs) as larger contributors to radicals and ozone production in the atmosphere. Atmospheric Chemistry and Physics.

[CR121] Wang X-T (2021). Trade-linked shipping CO_2_ emissions. Nature Climate Change.

[CR122] Wang X T (2021). Ship emissions around China under gradually promoted control policies from 2016 to 2019. Atmospheric Chemistry and Physics.

[CR123] Wang Y L (2021). Enhanced nitrite production from the aqueous photolysis of nitrate in the presence of vanillic acid and implications for the roles of light-absorbing organics. Environ. Sci. Technol..

[CR124] Wang Y L (2022). Decay kinetics and absorption changes of methoxyphenols and nitrophenols during nitratemediated aqueous photochemical oxidation at 254 and 313 nm. ACS Earth and Space Chemistry.

[CR125] Wang Z (2008). MICS-Asia II: Model inter-comparison and evaluation of acid deposition. Atmos. Environ..

[CR126] Wang Z L, Huang X, Ding A J (2018). Dome effect of black carbon and its key influencing factors: A one-dimensional modelling study. Atmospheric Chemistry and Physics.

[CR127] Wei N N, Fang B, Zhao W X, Wang C H, Yang N N, Zhang W J, Chen W D, Fittschen C (2020). Time-resolved laser-flash photolysis faraday rotation spectrometer: A new tool for total OH reactivity measurement and free radical kinetics research. Analytical Chemistry.

[CR128] Wei X L (2022). Technical note: Real-time diagnosis of the hygroscopic growth micro-dynamics of nanoparticles with Fourier transform infrared spectroscopy. Atmospheric Chemistry and Physics.

[CR129] Wu C H (2020). Measurement report: Important contributions of oxygenated compounds to emissions and chemistry of volatile organic compounds in urban air. Atmospheric Chemistry and Physics.

[CR130] Wu F, Song N, Hu T F, Ho S S H, Cao J J, Zhang D Z (2023). Surrogate atmospheric dust particles generated from dune soils in laboratory: Comparison with field measurement. Particuology.

[CR131] Wu F (2022). Saltation–sandblasting processes driving enrichment of water-soluble salts in mineral dust. Environmental Science & Technology Letters.

[CR132] Wu H J, Tang X, Wang Z F, Wu L, Li J J, Wang W, Yang W Y, Zhu J (2020). High-spatiotemporal-resolution inverse estimation of CO and NO_x_ emission reductions during emission control periods with a modified ensemble Kalman filter. Atmos. Environ..

[CR133] Wu L Q, Wang X M, Lu S H, Shao M, Ling Z H (2019). Emission inventory of semi-volatile and intermediate-volatility organic compounds and their effects on secondary organic aerosol over the Pearl River Delta region. Atmospheric Chemistry and Physics.

[CR134] Wu L Q, Ling Z H, Liu H, Shao M, Lu S H, Wu L L, Wang X M (2021). A gridded emission inventory of semi-volatile and intermediate volatility organic compounds in China. Science of the Total Environment.

[CR135] Wu L Q (2021). Roles of semivolatile/intermediate-volatility organic compounds on SOA formation over China during a pollution episode: Sensitivity analysis and implications for future studies. J. Geophys. Res.: Atmos.

[CR136] Xia C Z, Liu C, Cai Z N, Zhao F, Su W J, Zhang C X, Liu Y (2021). First sulfur dioxide observations from the environmental trace gases monitoring instrument (EMI) onboard the GeoFen-5 satellite. Science Bulletin.

[CR137] Xia M (2022). Pollution-Derived Br_2_ boosts oxidation power of the coastal atmosphere. Environ. Sci. Technol..

[CR138] Xia W W (2022). Double trouble of air pollution by anthropogenic dust. Environ. Sci. Technol..

[CR139] Xie X D, Wang T J, Yue X, Li S, Zhuang B L, Wang M H (2020). Effects of atmospheric aerosols on terrestrial carbon fluxes and CO_2_ concentrations in China. Atmospheric Research.

[CR140] Xie X D, Wang T J, Yue X, Li S, Zhuang B L, Wang M H, Yang X Q (2019). Numerical modeling of ozone damage to plants and its effects on atmospheric CO_2_ in China. Atmos. Environ..

[CR141] Xu B Q (2021). Compound-specific radiocarbon analysis of low molecular weight dicarboxylic acids in ambient aerosols using preparative gas chromatography: Method development. Environmental Science & Technology Letters.

[CR142] Xu B Q (2022). Large contribution of fossil-derived components to aqueous secondary organic aerosols in China. Nature Communications.

[CR143] Xu L, Du L, Tsona N T, Ge M F (2021). Anthropogenic effects on biogenic secondary organic aerosol formation. Adv. Atmos. Sci..

[CR144] Xu W, Ovadnevaite J, Fossum K N, Lin C S, Huang R J, Ceburnis D, O’Dowd C (2022). Sea spray as an obscured source for marine cloud nuclei. Nature Geoscience.

[CR145] Xu Z N (2021). Multifunctional products of isoprene oxidation in polluted atmosphere and their contribution to SOA. Geophys. Res. Lett..

[CR146] Xue T (2022). New WHO global air quality guidelines help prevent premature deaths in China. National Science Review.

[CR147] Yan C Q, Ma S X, He Q F, Ding X, Cheng Y, Cui M, Wang X M, Zheng M (2021). Identification of PM_2.5_ sources contributing to both Brown carbon and reactive oxygen species generation in winter in Beijing, China. Atmos. Environ..

[CR148] Yang H, Chen L, Liao H, Zhu J, Wang W J, Li X (2022). Impacts of aerosol–photolysis interaction and aerosol–radiation feedback on surface-layer ozone in North China during multi-pollutant air pollution episodes. Atmospheric Chemistry and Physics.

[CR149] Yang N N (2022). Optical-feedback cavity-enhanced absorption spectroscopy for OH radical detection at 2.8 µm using a DFB diode laser. Optics Express.

[CR150] Yang X P (2021). Observations and modeling of OH and HO_2_ radicals in Chengdu, China in summer 2019. Sci. Total Environ..

[CR151] Yang Y (2022). Abrupt emissions reductions during COVID-19 contributed to record summer rainfall in China. Nature Communications.

[CR152] Yang Z M, Du L, Li Y J, Ge X L (2022). Secondary organic aerosol formation from monocyclic aromatic hydrocarbons: Insights from laboratory studies. Environmental Science: Processes & Impacts.

[CR153] Yang Z M, Li K, Tsona N T, Luo X, Du L (2023). SO_2_ enhances aerosol formation from anthropogenic volatile organic compound ozonolysis by producing sulfur-containing compounds. Atmospheric Chemistry and Physics.

[CR154] Yang Z M, Xu L, Tsona N T, Li J L, Luo X, Du L (2021). SO_2_ and NH_3_ emissions enhance organosulfur compounds and fine particle formation from the photooxidation of a typical aromatic hydrocarbon. Atmospheric Chemistry and Physics.

[CR155] Ye C S (2021). Chemical characterization of oxygenated organic compounds in the gas phase and particle phase using iodide CIMS with FIGAERO in urban air. Atmospheric Chemistry and Physics.

[CR156] Ye Q (2021). High-resolution modeling of the distribution of surface air pollutants and their intercontinental transport by a global tropospheric atmospheric chemistry source–receptor model (GNAQPMS-SM). Geoscientific Model Development.

[CR157] Ye Q (2023). Uncertainties in the simulated intercontinental transport of air pollutants in the springtime from emission and meteorological inputs. Atmos. Environ..

[CR158] Yuan W (2020). Characterization of the light-absorbing properties, chromophore composition and sources of brown carbon aerosol in Xi’an, northwestern China. Atmospheric Chemistry and Physics.

[CR159] Zhang C X (2020). First observation of tropospheric nitrogen dioxide from the Environmental Trace Gases Monitoring Instrument onboard the GaoFen-5 satellite. Light: Science & Applications.

[CR160] Zhang G H (2019). Oxalate formation enhanced by Fe-containing particles and environmental implications. Environ. Sci. Technol..

[CR161] Zhang G H (2020). High secondary formation of nitrogen-containing organics (NOCs) and its possible link to oxidized organics and ammonium. Atmospheric Chemistry and Physics.

[CR162] Zhang G X (2022). Intercomparison of OH radical measurement in a complex atmosphere in Chengdu, China. Science of the Total Environment.

[CR163] Zhang H H (2022). Abundance and fractional solubility of aerosol iron during winter at a coastal city in northern China: Similarities and contrasts between fine and coarse particles. J. Geophys. Res.: Atmos.

[CR164] Zhang P, Chen T Z, Ma Q X, Chu B W, Wang Y H, Mu Y J, Yu Y B, He H (2022). Diesel soot photooxidation enhances the heterogeneous formation of H_2_SO_4_. Nature Communications.

[CR165] Zhang W Q (2020). Different HONO sources for three layers at the urban area of Beijing. Environ. Sci. Technol..

[CR166] Zhang X (2022). Influence of convection on the upper-tropospheric O_3_ and NO_x_ budget in southeastern China. Atmospheric Chemistry and Physics.

[CR167] Zhang Y-L (2022). A diurnal story of Δ^17^O(NO_3_^−^) in urban Nanjing and its implication for nitrate aerosol formation. npj Climate and Atmospheric Science.

[CR168] Zhang Z C (2022). Machine learning combined with the PMF model reveal the synergistic effects of sources and meteorological factors on PM_2.5_ pollution. Environ. Res..

[CR169] Zhao Y (2022). Decline in bulk deposition of air pollutants in China lags behind reductions in emissions. Nature Geoscience.

[CR170] Zheng B (2020). Satellite-based estimates of decline and rebound in China’s CO_2_ emissions during COVID-19 pandemic. Science Advances.

[CR171] Zheng B (2021). Mapping anthropogenic emissions in China at 1 km spatial resolution and its application in air quality modeling. Science Bulletin.

[CR172] Zheng M, Yan C Q, Zhu T (2020). Understanding sources of fine particulate matter in China. Philosophical Transactions of the Royal Society A: Mathematical, Physical and Engineering Sciences.

[CR173] Zhong X, Liu S C, Liu R, Wang X L, Mo J J, Li Y Z (2021). Observed trends in clouds and precipitation (1983–2009): Implications for their cause(s). Atmospheric Chemistry and Physics.

[CR174] Zhou J C (2020). Simultaneous measurements of the relative-humidity-dependent aerosol light extinction, scattering, absorption, and single-scattering albedo with a humidified cavity-enhanced albedometer. Atmospheric Measurement Techniques.

[CR175] Zhou J C (2022). Amplitude-modulated cavity-enhanced absorption spectroscopy with phase-sensitive detection: A new approach applied to the fast and sensitive detection of NO_2_. Analytical Chemistry.

[CR176] Zhu C-S, Qu Y, Huang H, Chen J, Dai W-T, Huang R-J, Cao J-J (2021). Black carbon and secondary brown carbon, the dominant light absorption and direct radiative forcing contributors of the atmospheric aerosols over the Tibetan Plateau. Geophys. Res. Lett..

[CR177] Zhu J, Chen L, Liao H (2022). Multi-pollutant air pollution and associated health risks in China from 2014 to 2020. Atmos. Environ..

[CR178] Zhu J L, Shang J, Zhu T (2021). A new understanding of the microstructure of soot particles: The reduced graphene oxide-like skeleton and its visible-light driven formation of reactive oxygen species. Environmental Pollution.

[CR179] Zhu J L, Shang J, Chen Y Y, Kuang Y, Zhu T (2020). Reactive oxygen species-related inside-to-outside oxidation of soot particles triggered by visible-light irradiation: Physicochemical property changes and oxidative potential enhancement. Environ. Sci. Technol..

[CR180] Zhu T, Dai S G (2005). Urban and regional air pollution complex. Series in Advances in Chemistry: Advances in Environmental Chemistry.

[CR181] Zhu T (2018). Air pollution in China: Scientific challenges and policy implications. National Science Review.

[CR182] Zhu T, Shang J, Zhao D F (2011). The roles of heterogeneous chemical processes in the formation of an air pollution complex and gray haze. Science China Chemistry.

[CR183] Zhu Y H (2020). Iron solubility in fine particles associated with secondary acidic aerosols in east China. Environmental Pollution.

[CR184] Zuo P J (2022). Stable iron isotopic signature reveals multiple sources of magnetic particulate matter in the 2021 Beijing sandstorms. Environmental Science & Technology Letters.

